# Origin of stabilization and destabilization in solid-state redox reaction of oxide ions for lithium-ion batteries

**DOI:** 10.1038/ncomms13814

**Published:** 2016-12-23

**Authors:** Naoaki Yabuuchi, Masanobu Nakayama, Mitsue Takeuchi, Shinichi Komaba, Yu Hashimoto, Takahiro Mukai, Hiromasa Shiiba, Kei Sato, Yuki Kobayashi, Aiko Nakao, Masao Yonemura, Keisuke Yamanaka, Kei Mitsuhara, Toshiaki Ohta

**Affiliations:** 1 Department of Green and Sustainable Chemistry, Tokyo Denki University, 5Senju Asahi-Cho, Adachi, Tokyo 120-8551, Japan; 2 Frontier Research Institute for Materials Science, Nagoya Institute of Technology, Gokiso-cho, Showa-ku, Nagoya, Aichi 466-8555, Japan; 3 Japan Science and Technology Agency (JST), PRESTO, 4-1-8 Honcho Kawaguchi, Saitama 332-0012, Japan; 4 Department of Applied Chemistry, Tokyo University of Science, 1-3 Kagurazaka, Shinjuku, Tokyo 162-8601, Japan; 5 Bio-engineering Lab., Riken, 2-1 Hirosawa, Wako, Satimata 351-0198, Japan; 6 Institute of Materials Structure Science (IMSS), High Energy Accelerator Research Organization (KEK), 1-1 Oho, Tsukuba, Ibaraki 305-0801, Japan; 7 Department of Materials Structure Science, Sokendai (The Graduate University for Advanced Studies), 203-1 Shirakata, Tokai, Ibaraki 319-1106, Japan; 8 SR Center, Ritsumeikan University, 1-1-1 Noji-Higashi, Kusatsu, Shiga 525-8577, Japan

## Abstract

Further increase in energy density of lithium batteries is needed for zero emission vehicles. However, energy density is restricted by unavoidable theoretical limits for positive electrodes used in commercial applications. One possibility towards energy densities exceeding these limits is to utilize anion (oxide ion) redox, instead of classical transition metal redox. Nevertheless, origin of activation of the oxide ion and its stabilization mechanism are not fully understood. Here we demonstrate that the suppression of formation of superoxide-like species on lithium extraction results in reversible redox for oxide ions, which is stabilized by the presence of relatively less covalent character of Mn^4+^ with oxide ions without the sacrifice of electronic conductivity. On the basis of these findings, we report an electrode material, whose metallic constituents consist only of 3*d* transition metal elements. The material delivers a reversible capacity of 300 mAh g^−1^ based on solid-state redox reaction of oxide ions.

## Abstract (web summary)

Energy storage by metal redox reactions sets strict limits on capacity in metal oxide cathode materials used in lithium-ion batteries. Here authors study stabilization of redox reactions at oxygen sites and demonstrate a cathode with a high reversible capacity enabled by the process.

Rechargeable lithium-ion batteries (LIBs) are widely used in our daily life because LIBs have the highest gravimetric/volumetric energy density among commercial energy storage devices. LIBs are used as a power source for zero emission electric vehicles and are expected to be used for grid energy storage^1^. LIBs are becoming a key technology enabling a shift from fossil fuel to renewable electric energy, which potentially realizes green and sustainable energy development in the future. Nevertheless, the possibility of further increase in the energy density is severely restricted because of unavoidable theoretical limits for positive electrodes, such as spinel-type oxides and iron phosphates.

In contrast to these materials, which are currently used in the commercial LIB, there is room for further increase in the energy density for layered oxides, such as LiCoO_2_ (ref. 2), LiNi_1/2_Mn_1/2_O_2_ (ref. 3), Li_2_MnO_3_ (ref. 4) and those derivatives^5^,^6^,^7^. Among them, Li_2_MnO_3_-based electrode materials have been extensively studied as positive electrode materials in the past decade^6^,^8^,^9^,^10^,^11^,^12^,^13^,^14^,^15^. The reaction mechanism of this material was a controversial subject for a long time. Since the oxidation state of manganese ions is tetravalent, oxidation of manganese ions beyond the tetravalent state is difficult. Instead of manganese ions, negatively charged anions, oxide ions (O^2−^), donate electrons on charge (electrochemical oxidation). However, oxidation of oxide ions results in partial loss of oxygen as an irreversible process, that is, decomposition reaction^8^. The loss of oxygen induces the formation of trivalent manganese ions on discharge (electrochemical reduction), leading to the unfavourable phase transition in the layered structure^11^. Nevertheless, it has been evidenced that reversible solid-state redox for oxide ions is possible for the Li_2_RuO_3_-based system, which essentially has the same crystal structure as Li_2_MnO_3_, and the contribution of oxide ions has been experimentally evidenced by using an arsenal of characterization techniques^14^ and theoretical method^16^. Very recently, the formation of peroxo-like dimers in Li_2−*x*_IrO_3_ has been experimentally visualized by transmission electron microscopy^17^ whereas recent experimental^18^ and theoretical^19^ studies have suggested that an isolated hole is formed on the charge process for lithium-excess electrode materials. Enrichment of lithium ions as the highly ionized cation results in a less covalent character for oxide ions, and thus the oxide ions are more easily oxidized compared with conventional oxides with late transition metals. Therefore, it has been proposed that the holes in the oxygen 2*p* orbital is effectively stabilized^18^,^19^.

The use of anion redox, especially oxide ions, is a crucial strategy to design and develop new electrode materials with high gravimetric/volumetric energy density for LIB. Reversible capacity of electrode materials is potentially further increased by the enrichment of lithium contents with less transition metals in the close-packed structure of oxide ions. Recently, our group has reported that Li_3_NbO_4_ (refs 20, 21) and Li_4_MoO_5_ (ref. 22), which have higher lithium contents than those of Li_2_MnO_3_ and Li_2_RuO_3_, are potentially utilized as host structures for a new series of high-capacity electrode materials. Similar concepts are also proposed in the literature^23^,^24^. Manganese-substituted Li_3_NbO_4_, Li_1.3_Nb_0.3_Mn_0.4_O_2_ (0.43Li_3_NbO_4_–0.57LiMnO_2_), delivers large reversible capacity (approximately 300 mAh g^−1^) with reversible solid-state redox reaction of oxide ions^20^. Similar to pentavalent niobium, a material with pentavalent antimony, Li_4_FeSbO_6_, has been recently reported^25^. Solid-state redox reaction of oxide ions is also activated in Li_4_FeSbO_6_, and a reductive coupling mechanism as an irreversible process has been evidenced in this system. As a non-rocksalt system, the use of Co-doped Li_2_O has been also proposed^26^. Although many articles now describes the anion redox for battery materials, the border between reversibility and irreversibility for the solid-state redox reaction of oxide ions remains unclear, and it is a critical point to understand the factors affecting reversibility of anion redox.

In this article, we answer these questions through systematic studies on Li_3_NbO_4_-LiMeO_2_ (Me=Fe, Mn and V) binary system. Reversibility of the solid-state redox reaction highly depends on the transition metal elements selected, which correlates with the formation of electrochemically unstable superoxide species because of charge transfer from oxidized oxide ions. Furthermore, on the basis of these findings, we demonstrate a niobium-free high-capacity positive electrode material, which effectively utilizes reversible solid-state redox reaction of oxide ions. These findings can potentially enable high-energy LIBs free of less abundant transition elements, such as cobalt and nickel ions.

## Results

### Synthesis and characterization of Li_3_NbO_4_-LiMeO_2_ system

**Figure 1:**
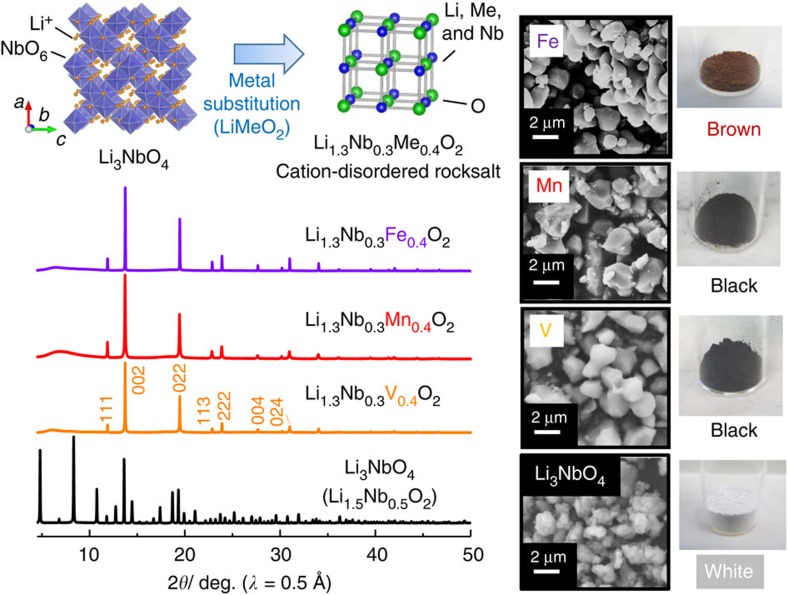
Crystal structures and particle morphology of Li_1.3_Nb_0.3_Me_0.4_O_2_. Synchrotron X-ray diffraction (SXRD) patterns of as-prepared 0.43 Li_3_NbO_4_–0.57 LiMeO_2_ (Me=Fe^3+^, Mn^3+^, and V^3+^) samples, which are reformulated as Li_1.3_Nb_0.3_Me_0.4_O_2_ based on the formulation of α-NaFeO_2_-type layered structure, are shown. SXRD patterns of the samples, Me=Fe^3+^ and Mn^3+^, were also shown in our previous work^20^. The samples crystallize into the cation disordered rocksalt structure with lattice parameters of 4.19 Å for Fe^3+^, 4.21 Å for Mn^3+^ and 4.17 Å for V^3+^. Structural analysis was carried out using RIETAN-FP (ref. 41). Substitution of Fe^3+^, Mn^3+^ and V^3+^ for Nb^5+^/Li^+^ results in the donation of *d* electrons in a conduction band, and thus colour of the samples changes from white for Li_3_NbO_4_. Data of Li_3_NbO_4_ (Li_1.5_Nb_0.5_O_2_) are also shown for comparison. Schematic illustrations of the crystal structures were drawn using the program VESTA (ref. 42).

Figure 1 summarizes characterization of Li_3_NbO_4_-LiMeO_2_ (Me=Fe, Mn and V) binary system by synchrotron X-ray diffraction (SXRD) and SEM. Li_3_NbO_4_ crystallizes into a cation-ordered rocksalt-type structure while a lack of *d* electrons in a conduction band results in an insulating character. Substitution of 3*d* transition metal ions for Nb/Li ions effectively induces conductive electrons, and colour of samples is also changed from white for Li_3_NbO_4_ to black for substituted samples with Mn^3+^ and V^3+^. Such 3*d* transition metals can accept electrons from oxide ions. However, a change in the crystal structure is also unavoidable, and formation of a cation-disordered rocksalt-type structure is found. Recently, electrode materials with the cation-disordered rocksalt-type structure, such as Li_1.211_Mo_0.467_Cr_0.3_O_2_ (ref. 27) and Li_1.33_V_0.67_O_1.33_F_0.67_ (ref. 28), have been reported as high-capacity electrode materials. Historically, such cation-disordered rocksalt phase had been regarded as electrochemically inactive as electrode materials because of a lack of the Li migration path in a bulk structure. Nevertheless, formation of percolating network for the Li-excess system (Li_1+*x*_Me_1−*x*_O_2_) opens the path for Li migration in the cation-disordered rocksalt-type structure^27^.

### Electrochemistry of Li_3_NbO_4_-LiMeO_2_ system in half-cells

**Figure 2:**
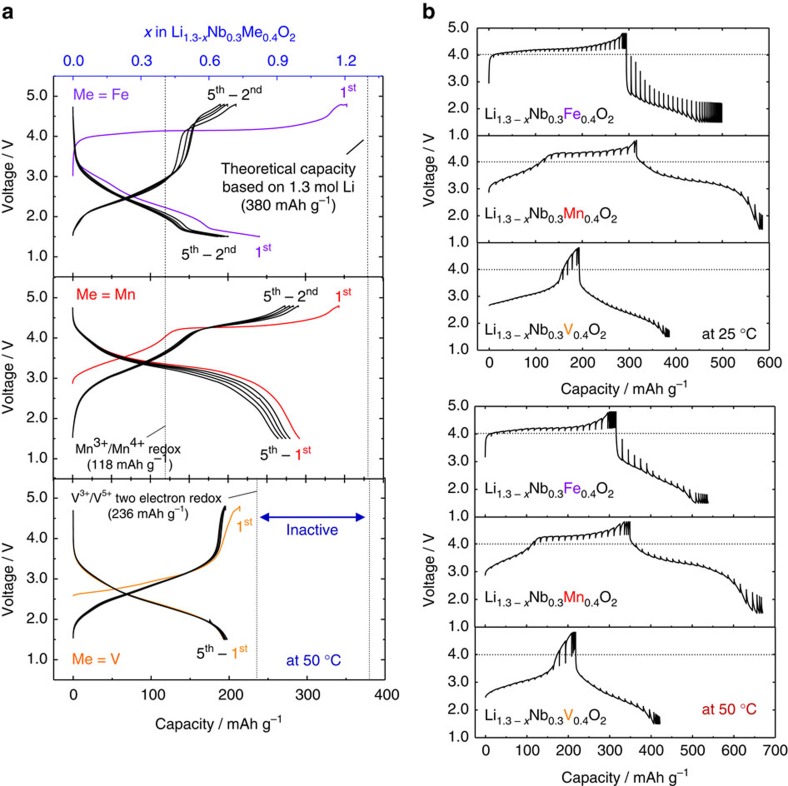
Electrochemical properties of Li_1.3_Nb_0.3_Me_0.4_O_2_ in Li cells. (**a**) Galvanostatic oxidation/reduction curves of Li_1.3_Nb_0.3_Me_0.4_O_2_ (Me=Fe^3+^, Mn^3+^ and V^3+^) samples in Li cells at 10 mA g^−1^ at 50 °C. Quasi open circuit voltage (QOCV) of the samples measured by galvanostatic intermittent titration technique (GITT) is also plotted in (**b**); charge for 1 h at 10 mA g^−1^ at room temperature and 50 °C and rest for 5 h.

Although as-prepared samples with primary particle size of 2–3 μm show insufficient electrode performance^20^, mechanical ball milling with carbon (reduction of particle size with uniform mixing with carbon is achieved in Supplementary Fig. 1) effectively improves the electrode performance of the samples in Li cells. Electrode performance is further improved at elevated temperature (50 °C), and three cation-disordered rocksalt samples, Li_1.3_Nb_0.3_Me_0.4_O_2_ (Me=Fe^3+^, Mn^3+^ and V^3+^), deliver large reversible capacities in Li cells, as shown in Fig. 2. It is noted that three Li_3_NbO_4_-based samples with different 3*d* transition metals show quite different electrochemical behaviour. The Fe system shows a large initial charge (oxidation) capacity of 350 mAh g^−1^ that is quite close to that of the theoretical capacity (383 mAh g^−1^) as defined by the extraction of all Li^+^ ions (1.3 moles) from the crystal lattice. However, a clear change in a voltage profile is found on the second charge process, which is totally different from the first charge process. A well-defined voltage plateau is observed on the initial charge, but an S-shaped profile is noted after first discharge process. Polarization is small for the S-shaped profile centred at 2.5 V, which will be discussed in the later section. The voltage plateau is not observed for the V system, and the sample shows S-shaped profile from the initial charge process. The observed reversible capacity is comparable to that of a theoretical capacity based on V^3+^/V^5+^ two-electron redox (236 mAh g^−1^) and is much smaller than that expected from lithium contents in the structure. The Mn system delivers a large discharge capacity of 300 mAh g^−1^ with the appearance of voltage plateau as reported in our literature^20^. Capacity retention of the samples is compared in Supplementary Fig. 2 at room temperature. Although the Mn system shows large reversible capacity, the V and Fe systems show better capacity retention in Li cells. Quasi-open-circuit voltage (QOCV) of the samples at 25 and 50 °C are also compared in Fig. 2b. The Fe system shows quite small polarization with flat QOCV at 4 V on charge whereas huge polarization (> 1.5 V) is observed on discharge at 25 °C. The polarization is significantly reduced at 50 °C. The V system shows the smallest polarization on charge/discharge with an S-shaped voltage profile as expected from galvanostatic curves (Fig. 2a). Two different regions are observed for the Mn system on charge; relatively small polarization for the sloping region from 3 to 4 V and slightly large polarization for the plateau region at 4.2 V. On the discharge process, two regions are not distinguished and a continuous S-shaped profile on QOCV is observed. Such behaviour originates from the hysteresis of oxidation/reduction reaction for solid-state redox of oxide ions.

### Charge compensation mechanisms in Li_3_NbO_4_-LiMeO_2_ system

**Figure 3:**
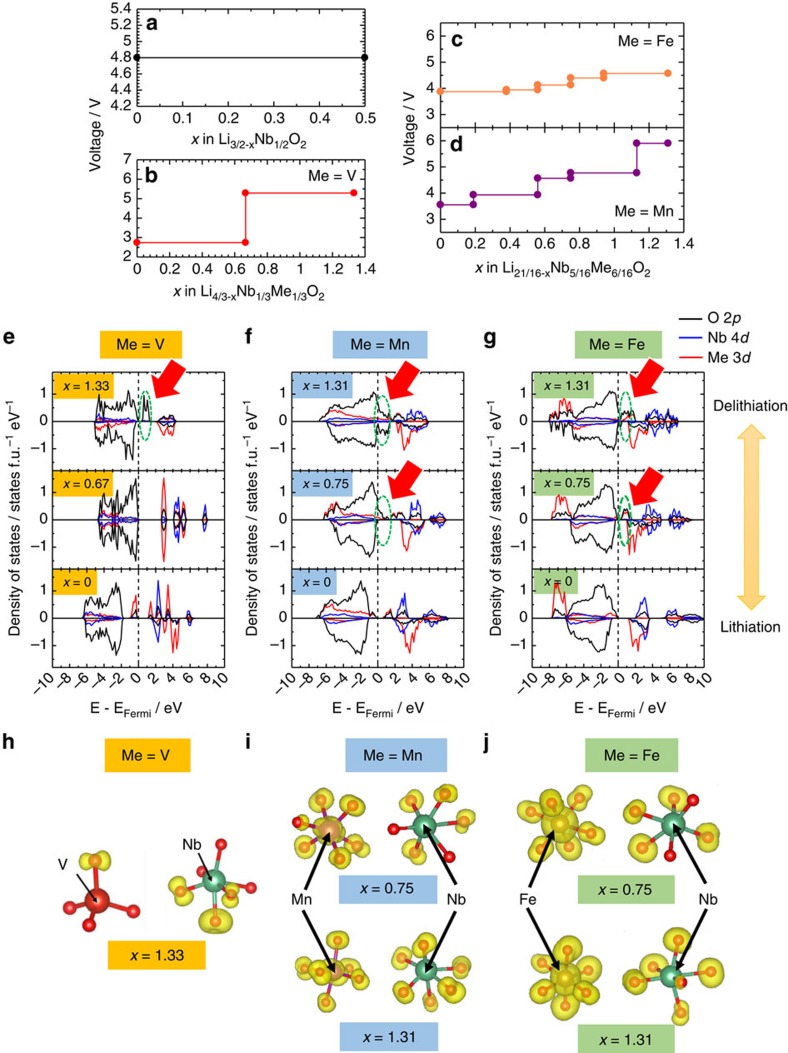
Summary of DFT calculations for Li-Nb-Me-O system. Calculated voltage profiles for (**a**) Li_3_NbO_4_ (Li_1.5_NbO_2_), (**b**) Li_4/3_V_1/3_Nb_1/3_O_2_, (**c**) Li_21/16−*x*_Fe_6/16_Nb_5/16_O_2_ and (**d**) Li_21/16−*x*_Mn_6/16_Nb_5/16_O_2_, and partial density of states (PDOS) are also shown in **e**–**g**. PDOS of Li_3−*x*_NbO_4_ is shown in Supplementary Fig. 15. Transition metal ions are mainly oxidized at an early stage of Li removal, whereas oxide ions are responsible for charge compensation at a late stage. The highest voltage is seen for M=Fe, followed in order by Mn and V, at an early stage of Li removal. Fe 3*d* orbital strongly hybridized with O 2p orbital at *x*=0.75 while weak hybridization is observed for the Mn system. V 3*d* orbital is localized at above O 2p level. These are supported by the PDOS diagrams and calculated partial charge density of electron at the hole state near to Fermi level, as shown in **h**–**j**).

Three different samples show quite different electrochemical behaviour in Li cells, as shown in Fig. 2. Such behaviour is expected to originate from differences in charge compensation mechanisms depending on the 3*d* transition metals (Fe, Mn and V) in the Li_3_NbO_4_ framework. Charge compensation mechanisms in Li cells were, therefore, examined using a combination of different characterization methods: synchrotron SXRD, hard/soft X-ray absorption spectroscopy (XAS) and X-ray photoelectron spectroscopy (XPS) with the assist of density functional theory (DFT) calculations. Results are described in detail in Supplementary Figs 3–10, and DFT calculations in Fig. 3, Supplementary Figs 11–15 and Supplementary Table 1.

**Figure 4:**
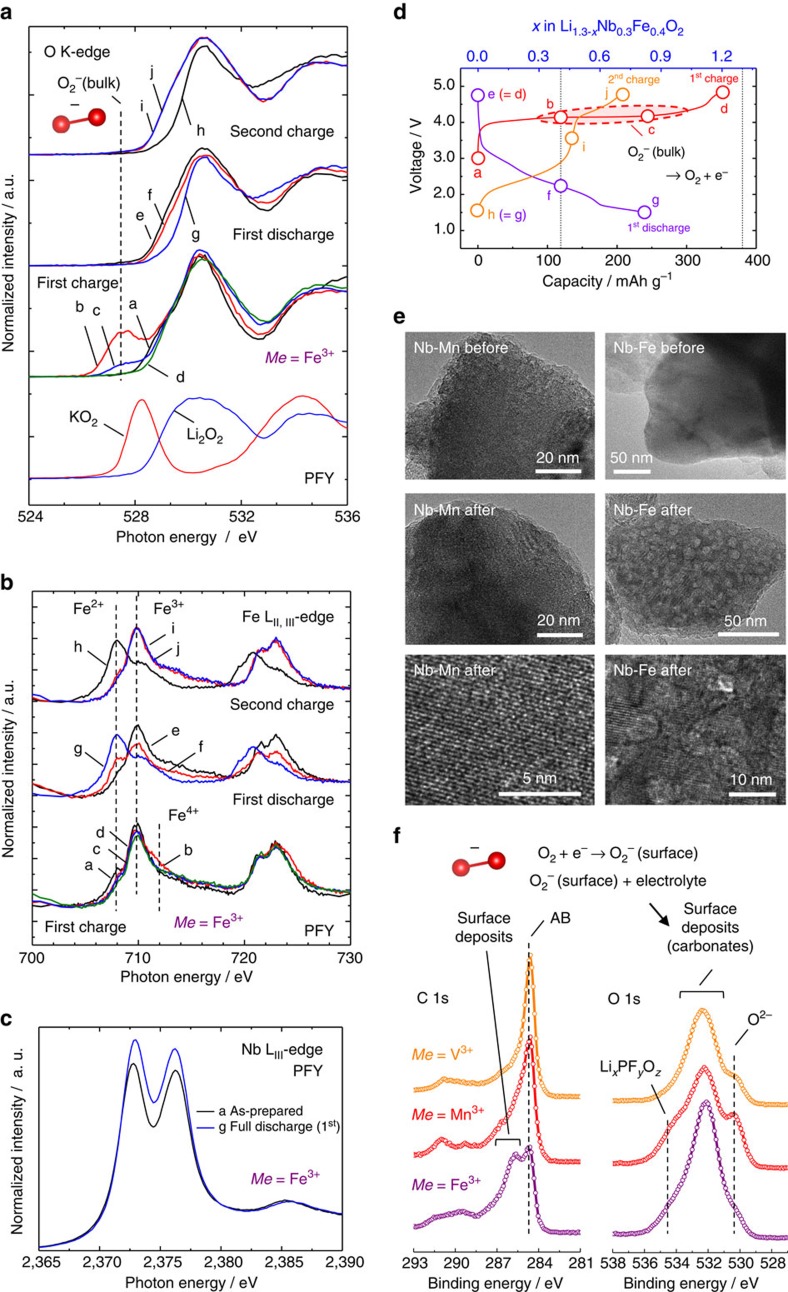
Changes in electronic structures for Li_1.3−*x*_Nb_0.3_Fe_0.4_O_2−*δ*_ on initial charge/discharge and second charge. Changes in the O K-edge (**a**) Fe L_II, III_-edge XAS spectra (**b**), Nb L_III_-edge XAS spectra (**c**), and the points where XAS spectra have been collected in (**d**). XAS spectra of KO_2_ (superoxide) and Li_2_O_2_ (peroxide) are also shown in (**a**) for comparison. Niobium is not responsible for charge compensation process (other data sets of Nb are shown in Supplementary Fig. 10). (**e**) TEM images of Li_1.3_Nb_0.3_Fe_0.4_O_2_ and Li_1.3_Nb_0.3_Mn_0.4_O_2_ particles before and after the electrochemical cycle at 50 °C. Oxygen loss for the Fe system results in the formation of nanosized grains in the single particle, and not for the Mn system. Lattice fringes are clearly observed after electrochemical cycle for the Mn system. (**f**) Surface structures observed by X-ray photoelectron (XPS) spectroscopy after discharge to 1.5 V. Oxygen molecules released on charge result in the electrochemical reduction on discharge, leading to the formation of surface deposits because of chemical reaction between active superoxide with electrolyte, as shown in our previous literature^11^.

Important findings are summarized as follows: (1) In the Mn system, reversible oxidation of oxide ions, coupled with Mn^3+^/Mn^4+^ redox, is realized as reported in our previous work^20^. (2) In the V system, V^3+^/V^5+^ two-electron redox is active, but the oxidation of oxide ions is not evidenced. (3) In the Fe system, the formation of superoxide (O_2_^–^) (ref. 29) is observed from the measurement of O K-edge XAS, which is further supported by the DFT study with COOP analysis (Supplementary Fig. 14). Since the most significant changes on charge/discharge curves were observed for the Fe system, changes in soft XAS spectra were examined on the initial cycle and second charge at 50 °C (Fig. 4). Formation of superoxide is further pronounced on charge at 50 °C, and superoxide is stabilized in the bulk of particle rather than the surface (Supplementary Fig. 9). However, superoxide species are electrochemically oxidized and decomposed by further charge. The superoxide species disappears as seen in the O K-edge spectra after charge to 4.8 V. This process inevitably results in the oxygen loss and structural reconstruction process (the latter is also supported by transmission electron microscopy as the formation of nanosize grains, as shown in Fig. 4e). Origin of the formation of superoxide is discussed in the later section. The oxygen loss results in the reduction of Fe^3+^ to Fe^2+^ and accumulation of surface deposits on discharge. The voltage plateau is significantly shortened on the second charge, and the S-shaped voltage profile centred at 2.5 V is observed with relatively small polarization. This reaction mainly originates from Fe^2+^/Fe^3+^ redox, as shown in Fig. 4b, and the formation of superoxide is not found in the second charge. In the conventional Li-excess system, Li_1+*x*_Ni_*y*_Co_*z*_Mn_(1–*x*–*y*–*z*)_O_2_, a clear voltage plateau is observed at 4.6 V at the initial charge process, but not for the second charge. An S-shaped voltage profile without the plateau region is observed from the second charge with activated Mn^3+^/Mn^4+^ redox^8^,^9^,^11^,^12^. The reversible contribution of oxide ions for charge compensation is less pronounced in these systems. Oxygen loss on charge is further supported by XPS (Fig. 4f and Supplementary Fig. 8). Oxygen molecules released in the cells are electrochemically reduced on discharge, leading to the formation of superoxide (on the surface of electrode), which further reacts with electrolyte^11^,^30^,^31^. This process is clearly evidenced, especially for the Fe system, as the accumulation of surface deposits on AB and active materials (Fig. 4f and Supplementary Fig. 8).

In contrast, for Li_1.3_Nb_0.3_Mn_0.4_O_2_, reversible changes in O K-edge XAS spectra are observed (Supplementary Fig. 6). Moreover, a clear voltage plateau is observed even in the ‘second' charge (Fig. 2), indicating that solid-state redox reaction is a reversible process. Note that DFT calculations also support these findings. Formation energy of the charged Mn system is energetically stable, but segregation (decomposition) to LiFeO_2_ and LiNbO_3_ accompanying O_2_ gas evolution is energetically preferable for the Fe system (see equations (s3) and (s4) in Supplementary Methods). Nevertheless, the plateau gradually becomes shorter in the continuous cycles probably because of the increase in polarization. Cyclability as the electrode material is expected to be further improved through the optimization of battery components, for example, electrolyte, binder, surface coating of particle and so on, and controlling charge conditions, as shown in our previous work^20^.

A question remains in the V system. Why are not oxide ions experimentally oxidized in this system? Theoretical prediction in Fig. 3 indicates that (1) two-electron redox of V^3+^/V^5+^ occurs coupled with vanadium migration to tetrahedral sites, which is consistent with experimental finding (see the Supplementary Methods for theoretical and experimental evidences) and (2) oxidation of oxide ions is also possible in the V system, which was not experimentally observed on charge to 4.8 V, as shown in Fig. 2. This inconsistency for the oxide ion redox simply originates from the difficultly of the electron transfer from oxide ions to V^5+^ for the fully charged state, namely kinetic limitation. DFT calculation clearly supports this fact. Oxide ions are theoretically oxidized, but the hole induced in oxygen 2*p* orbital is isolated in the structure, as shown in Fig. 3. Vanadium ions in Li_0.5_Nb^5+^_0.3_V^5+^_0.4_O_2_ (fully charged state), therefore, cannot transfer electrons from oxide ions and oxidation of oxide ions in the V system is kinetically restricted. Similar situation is observed for Li_3−*x*_NbO_4_ as a model material. Calculated voltage by DFT study is estimated to be 4.8 V for Li_3_NbO_4_ (Li_3/2_Nb_1/2_O_2_, Fig. 3a). Corresponding partial density of states diagram and partial electron density are also shown in Supplementary Fig. 15. Oxidation of O^2−^ ions is indicated during delithiation by DFT study since creation of O 2*p* hole level is clearly visible just above Fermi level at *x*=0.5 in Li_3/2−*x*_Nb_1/2_O_2_. However, holes are completely isolated in the structure, and thus electron transfer between O 2*p* and Nb^5+^ is kinetically limited as observed in the experimental study^20^.

### Design of high-capacity electrode materials with anion redox

As shown in this article, oxidation of oxide ions is not difficult, and only electron transfer from oxide ions to 3*d* transition metals is a necessary condition. However, the stabilization of oxidation reaction of oxide ions is not easy. In many materials, oxidation of oxide ions is possible, but this process induces oxygen loss, as in the cases of Li_2_MnO_3_ (ref. 4), Li_4_FeSbO_6_ (ref. 32), Li_4_NiMoO_6_ (ref. 22) and so on^8^,^24^. The reversible contribution of oxide ions for charge compensation has been reported only in limited electrode materials, for example, Li_2_Ru_1−*y*_Me_*y*_O_3_ (Me=Ru, Sn and Ti) (refs 14, 33), and the Nb-Mn system shown in this study. In general, peroxide/superoxide species are stabilized for sp elements without valence electrons (K^+^, Ca^2+^ and so on ) and d^10^ closed-shell (Zn^2+^, Cd^2+^ and so on). In contrast, transition metal oxides often decompose these species. One typical example is a disproportionation reaction of H_2_O_2_ catalysed by MnO_2_. This reaction is triggered by electron transfer between peroxide ions and surface manganese ions. Nb^5+^ also has no valence electron, and therefore a similar role is anticipated with the sp elements. Among 3*d* transition metal elements, Ti^4+^ has a similar electronic configuration with Nb^5+^. These ions most probably screen off electrons of unstable ‘oxidized' oxide ions and thus suppresses charge transfer. In addition, Nb^5+^ and Ti^4+^ are highly ionized ions compared with late transition metal ions with oxide ions, and thus the mixing between metal *d* orbital and oxygen 2*p* orbital is less pronounced. Therefore, similar to the lithium enrichment^18^,^19^, a character of oxide ions becomes more ionic (approaches two minus as a net charge) because of electron donation from Nb and Ti, and this fact would be beneficial to stabilize the oxidation of oxide ions.

**Figure 5:**
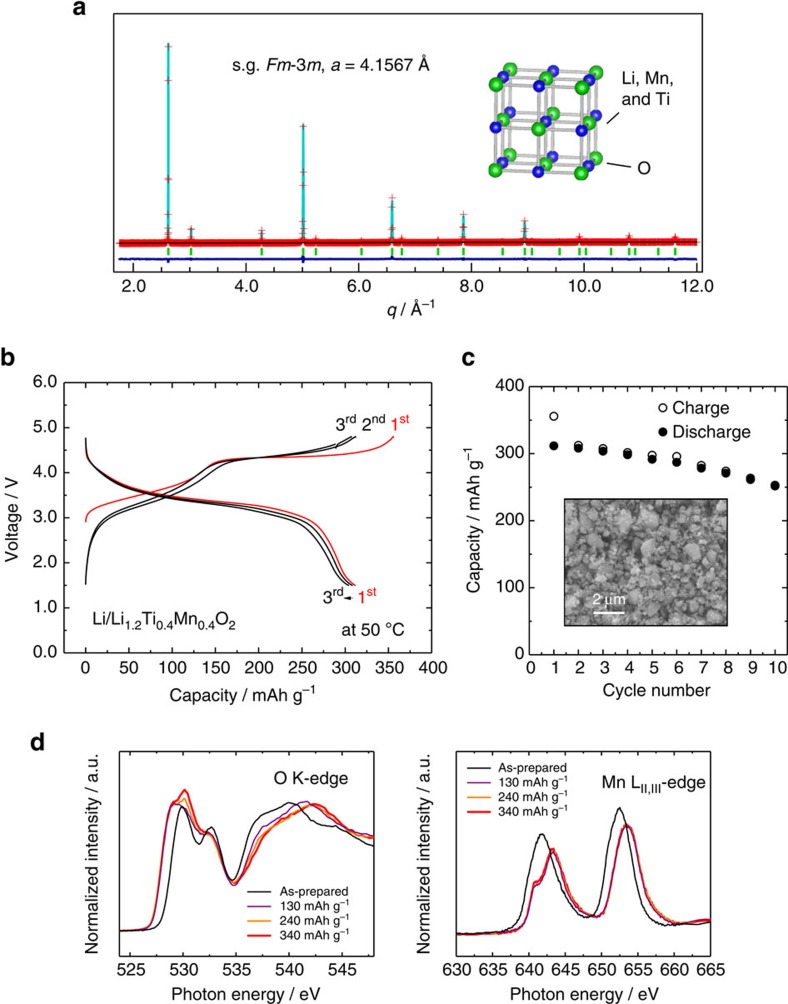
Li_1.2_Ti_0.4_Mn_0.4_O_2_ with a cation disordered rocksalt structure. (**a**) Neutron diffraction pattern of the as-prepared sample; (**b**) charge/discharge curves in a Li cell at a rate of 5 mA g^−1^ at 50 °C; (**c**) discharge capacity retention and an SEM image of the ball-milled sample and (**d**) changes in O K-edge and Mn L_II, III_-edge XAS spectra on charge in Li cells. The samples used in (**d**) were prepared at 50 °C in Li cells.

To test this hypothesis, a binary system of Li_2_TiO_3_−LiMnO_2_ has been examined. One to one composition between Li_2_TiO_3_ and LiMnO_2_ has been synthesized, which is reformulated as Li_1.2_Ti_0.4_Mn_0.4_O_2_. A mole fraction of Mn was adjusted to be the same with Li_1.3_Nb_0.3_Mn_0.4_O_2_. Similar to Li_3_NbO_4_, the cation-disordered rocksalt phase is obtained for Li_1.2_Ti_0.4_Mn_0.4_O_2_ as a single phase, and uniform size (2–3 μm) of primary particles is found by SEM (Supplementary Fig. 16). A result of structural analysis by neutron scattering is also provided in Fig. 5a and Supplementary Table 2. A mole fraction of Li in the cation-disordered rocksalt phase is inevitably lowered by the use of Ti^4+^. This fact results in the increase in penalty for percolating Li migration in the structure^27^,^34^. However, percolation probability of electron migration through the bonding between oxide ions and manganese ions (40% in the structure) is comparable with that of Li_1.3_Nb_0.3_Mn_0.4_O_2_. Li_1.2_Ti_0.4_Mn_0.4_O_2_ was mixed with 10 wt% acetylene black (HS-100, Denka Co. Ltd) and ball-milled to enhance the electrode performance. Thus prepared sample shows a large reversible capacity, as shown in Fig. 5b, and the Nb-free sample delivers more than 300 mAh g^−1^ at 50 °C. A voltage profile of Li_1.2−*x*_Ti_0.4_Mn_0.4_O_2_ quite resembles that of Li_1.3−*x*_Nb_0.3_Mn_0.4_O_2_. Available energy density of Li_1.2−*x*_Ti_0.4_Mn_0.4_O_2_ exceeds 1,000 mWh g^−1^ as a positive electrode material. Capacity retention is much better than that of pure Li_2_MnO_3_ (ref. 35) and slightly improved in comparison to Li_1.3−*x*_Nb_0.3_Mn_0.4_O_2_, as shown in Fig. 5c. To examine whether oxide ions are oxidized and stabilized in Li_1.2−*x*_Ti_0.4_Mn_0.4_O_2_, reaction mechanisms were examined by soft XAS with fluorescence yield in the synchrotron facility^36^. On the initial charge, Mn^3+^ is oxidized to Mn^4+^ for the slope region to 4 V as evidenced from Mn L-edge XAS spectra, and this fact also influences the profile of O K-edge XAS spectra (Fig. 5d). No change in Mn L-edge XAS spectra is observed for the plateau region at 4.2 V. A change in electronic structures is also not evidenced for Ti L-edge XAS spectra (Supplementary Fig. 17). Similar to Li_1.3−*x*_Nb_0.3_Mn_0.4_O_2_, a new peak appears at ca. 530 eV, (see Supplementary Fig. 6 for more details), which is further intensified as increase in the charge capacity for the plateau region, and the formation of superoxide is not evidenced. Similar observation is also noted for the recent study for the conventional Li-excess system^18^, but the change is more clearly pronounced in the Ti-Mn system. Although the energy of the new peak is consistent with that of Li_2_O_2_, further study is needed to understand the factor affecting the profile of XAS spectra. Nevertheless, the possibility of the formation of superoxide species can be excluded. Such change potentially originates from two possibilities: the formation of isolated holes^18^,^19^ and/or *σ*-hybridization as theoretically proposed in the Ru-Sn system after delithiation^19^. An almost identical profile with the pristine sample is observed after discharge to 1.5 V, suggesting the reversible process. Very recently, electrode performance and changes in O K-edge XAS spectra on charge have been reported for Li_1.19_Ti_0.38_Fe_0.57_O_2_ (ref. 37) and Li_1.42_Mo_0.29_Fe_0.29_O_2_ (ref. 38). Similar to Li_1.3−*x*_Nb_0.3_Fe_0.4_O_2_ clear evidence for the formation of superoxide and large polarization on charge/discharge have been reported for both samples.

**Figure 6:**
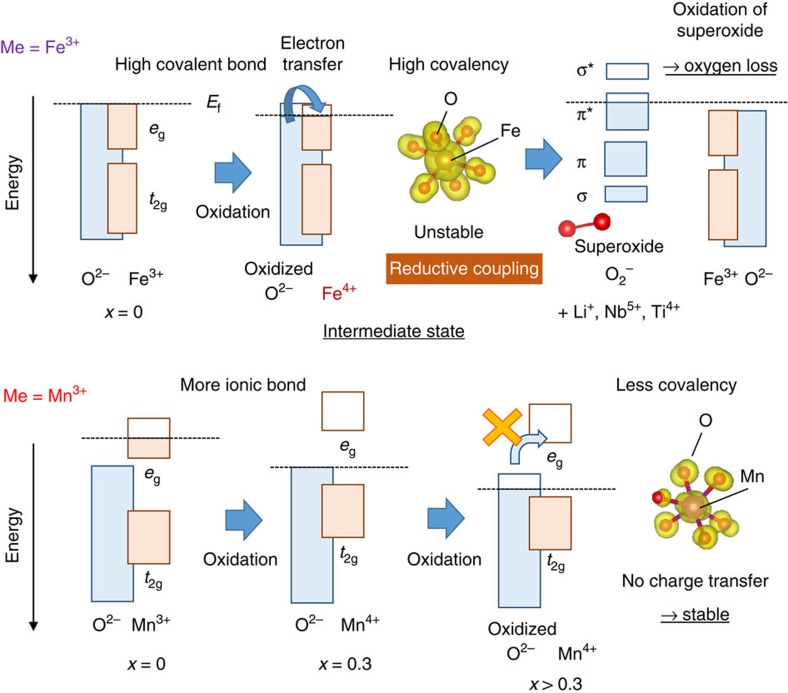
Proposed stabilization/destabilization mechanisms for solid-state redox reaction of oxide ions. Charge compensation mechanisms on charge in Li cells are compared for Li_1.3−*x*_Nb_0.3_Me_0.4_O_2_ (Me=Fe^3+^ and Mn^3+^). A similar stabilization mechanism is anticipated for Li_1.2−*x*_Ti_0.4_Mn_0.4_O_2_.

The selection of 3*d* transition metals, which accept electrons from oxide ions on charge, is an essential key to determine whether to stabilize oxidation of oxide ions (the formation and stabilization of isolated holes as proposed in the literature^18^,^19^) or to form unstable superoxide-like species. Stabilization/destabilization mechanisms for Mn/Fe are proposed in Fig. 6. For the case of Nb(Ti)-Fe, the DFT data in Fig. 3 suggest that both oxide and iron ions are simultaneously oxidized on charge (namely oxidation of high covalent Fe-O bond). However, only trace amount of Fe^4+^ is experimentally found in the Fe L-edge spectra. This fact suggests that electron transfer occurs from ‘oxidized' oxide ions (or a hole induced in O 2*p*) to Fe^4+^ because energy levels of both ions are similar for each other, namely highly covalent. Electrons are donated from oxidized oxide ions to neighbouring Fe^4+^ ions, leading to the formation of superoxide and Fe^3+^. This process is also called as the reductive coupling^14^,^16^. The formation of superoxide is further supported by the DFT study with COOP analysis (Supplementary Fig. 14). Such superoxide species would be stabilized by coupling with Li^+^ and Nb^5+^ (Ti^4+^) ions. Similar results are expected for Ni^3+/4+^ and Co^3+/4+^ with heavily hybridized characters for oxide ions near the Fermi level and indeed oxygen loss is experimentally observed for Li-excess electrode materials with these elements. In contrast, oxide ions do not donate electrons to less covalent Mn^4+^ with the *d*^3^ configuration (as the high-spin configuration) associated with energy gap between filled *t*_2g_ and empty *e*_g_ bands, as shown in Figs 3 and 6. Ru^5+^ also have a similar electronic configuration. Since the energy level of *t*_2*g*_ orbital for Mn^4+^ is high enough than that of Fermi level, oxide ions are solely oxidized on further oxidation. The presence of electrons in *t*_2*g*_ orbital is also essential without the sacrifice of electronic conductivity in bulk. Moreover, this reaction is further stabilized by the presence of Nb^5+^ and Ti^4+^, which donates electrons to oxide ions because of high ionic characters as cations and completely suppresses charge transfer from oxidized oxide ions.

In conclusion, the use of solid-state redox reaction of oxide ions is an effective strategy to further increase energy density of LIBs. We have demonstrated that the stabilization of redox reaction for oxide ions is possible using a combination of only 3*d* transition metals as the metallic constituents. This serves as a significant proof-of-concept towards practical applications. We expect that by relaxing the constraints posed on materials design by the conventional concept of transition metal redox, many new positive electrode materials with high capacity will appear, similar to Li_1.2_Ti_0.4_Mn_0.4_O_2_.

## Methods

### Synthesis of materials

Li_3_NbO_4_ was prepared by solid-state reaction from stoichiometric amounts of Li_2_CO_3_ (> 98.5%; Kanto Kagaku) and Nb_2_O_5_ (99.9%; Wako Pure Chemical Industries) at 950 °C for 24 h in air. Li_1.3_Nb_0.3_Me_0.4_O_2_ (Me=Fe^3+^, Mn^3+^ and V^3+^) samples were prepared from Li_2_CO_3_, Nb_2_O_5_ and precursors containing each transition metal: Mn_2_O_3_, Fe_2_O_3_ (99.9%; Wako Pure Chemical Industries), V_2_O_3_ (98%; Sigma-Aldrich Japan). Mn_2_O_3_ was obtained by heating of MnCO_3_ (Kishida Chemical) at 700 °C for 12 h. The precursors were thoroughly mixed by wet mechanical ball milling and then dried in air. Thus obtained mixtures of the samples were pressed into pellets. The pellets were heated at 900 °C for 12 h in air (Fe^3+^) or inert atmosphere (Mn^3+^ and V^3+^). The samples were stored in an Ar-filled glove box until use.

Li_1.2_Ti_0.4_Mn_0.4_O_2_ was prepared from Li_2_CO_3_, TiO_2_ (Anatase, 98.5%; Wako Pure Chemical Industries), and Mn_2_O_3_. The precursors were thoroughly mixed by wet mechanical ball milling and the mixture was heated at 900 °C for 12 h in inert atmosphere. Particle morphology of the samples was observed using a scanning electron microscope (JCM-6000, JEOL) with acceleration voltage of 15 keV.

### Electrochemical characterization

Electrode performance of the samples was examined for the carbon composite samples prepared by ball-milling. As-prepared Li_1.3_Nb_0.3_Me_0.4_O_2_ (Me=Fe^3+^, Mn^3+^ and V^3+^) were mixed with acetylene black by using a planetary ball mill (PULVERISETTE 7; FRITSCH) at 300 r.p.m with a zirconia container and balls. The samples used in Fig. 2, Supplementary Figs 2 and 8 were mixed with 10 wt% carbon. Composite electrodes consisted of 76.5 wt% active materials, 13.5 wt% acetylene black and 10 wt% poly(vinylidene fluoride), pasted on aluminium foil as a current collector. Rest of the data were collected using the samples with 20 wt% carbon. Composite electrodes consisted of 72 wt% active materials, 18 wt% acetylene black and 10 wt% poly(vinylidene fluoride). Metallic lithium (Honjo Metal) was used as a negative electrode. The electrolyte solution used was 1.0 mol dm^−3^ LiPF_6_ dissolved in ethylene carbonate:dimethyl carbonate (1:1 by volume) (Kishida Chemical). A polyolefin microporous membrane was used as a separator. R2032-type coin cells (Hosen Corp.) or TJ-AC (Tomcell Japan) were assembled in the Ar-filled glove box. The cells were cycled at a rate of 10 mA g^−1^ at room temperature or 50 °C.

### Materials characterization

Soft X-ray absorption (XAS) spectra were collected at BL-11 (O K-edge and Me L_II, III_-edges) in the synchrotron facility of Ritsumeikan University (Synchrotron Radiation Center)^36^. The absorption spectra were collected with fluorescence yield and electron yield modes. Similar to the measurements for hard XAS, the samples were prepared in the Ar-filled glove box, and thus prepared samples were set on the spectrometer using a laboratory-made transfer vessel without air exposure. Normalization of the XAS spectra was carried out using the program code IFEFFIT (ref. 39). The postedge background was determined using a cubic spline procedure.

XPS measurements were carried out with VG ESCALAB 250 spectrometer (Thermo Fisher Scientific K.K.) using monochromatized Al Kα X-ray radiation (1486.6 eV). The system was operated at 15 kV and 200 W. The base pressure of the analysis chamber was less than 10^−8^ Pa. These characterizations were carried our using a laboratory-made transfer vessel to avoid the sample exposure to moisture/air.

transmission electron microscopy observation was conducted by using JEM-ARM200F (JEOL) operated at 200 keV. The samples were dispersed in dimethyl carbonate and then supported on a copper grid.

A neutron diffraction pattern was collected at BL09 (SPICA) in the Material and Life science Facility (MLF) of the Japan Proton Accelerator Research Complex (J-PARC) (ref. 40).

### Data availability

The data supporting the main findings of this study are available from the corresponding authors on request.

## Additional information

**How to cite this article:** Yabuuchi N, *et al*. Origin of stabilization and destabilization in solid-state redox reaction of oxide ions for lithium-ion batteries. *Nat. Commun.* **7,** 13814 doi: 10.1038/ncomms13814 (2016).

**Publisher's note:** Springer Nature remains neutral with regard to jurisdictional claims in published maps and institutional affiliations.

## Supplementary Material

Supplementary Information Supplementary Figures, Supplementary Tables, Supplementary Methods and Supplementary References
