# Supplementary material for: Origin of stabilization and destabilization in solid-state redox reaction of oxide ions for lithium-ion batteries
Source: Nat Commun. 2016 Dec 23;7:13814. doi: 10.1038/ncomms13814 (PMC5196437; doi:10.1038/ncomms13814)
Supplement: Supplementary Information — Supplementary Figures, Supplementary Tables, Supplementary Methods and Supplementary References [file ncomms13814-s1.pdf]

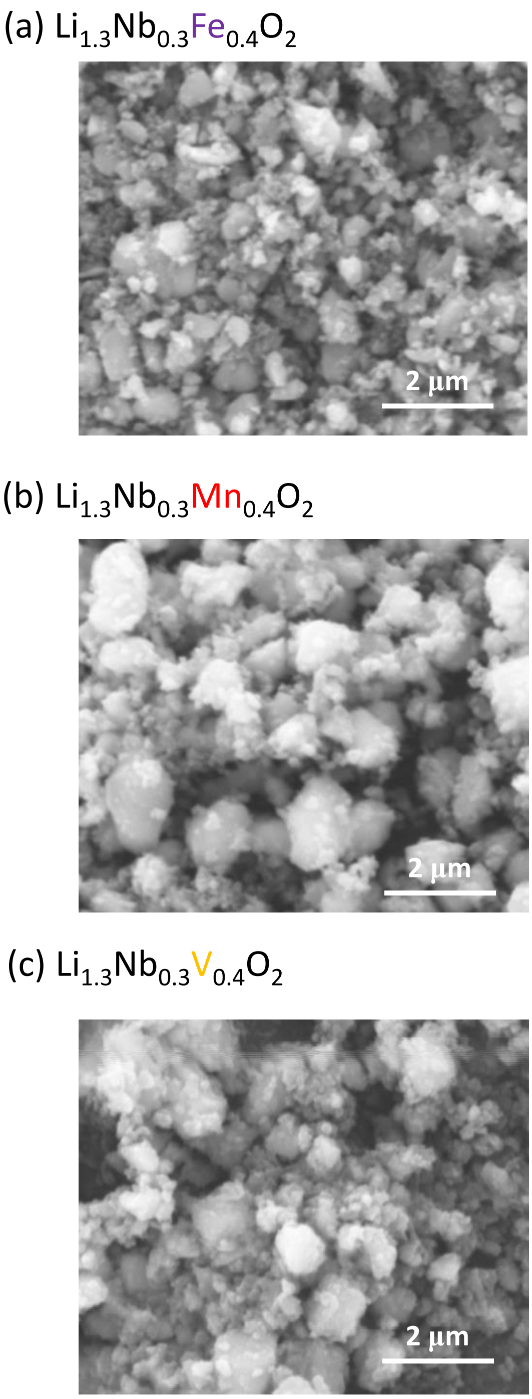

Supplementary Figure 1. SEM images of $Li_{1.3}Nb_{0.3}Me_{0.4}O_2$ (Me = Fe^{3+}, Mn^{3+}, and V^{3+}) samples after ball milling with 10 wt% acetylene black.

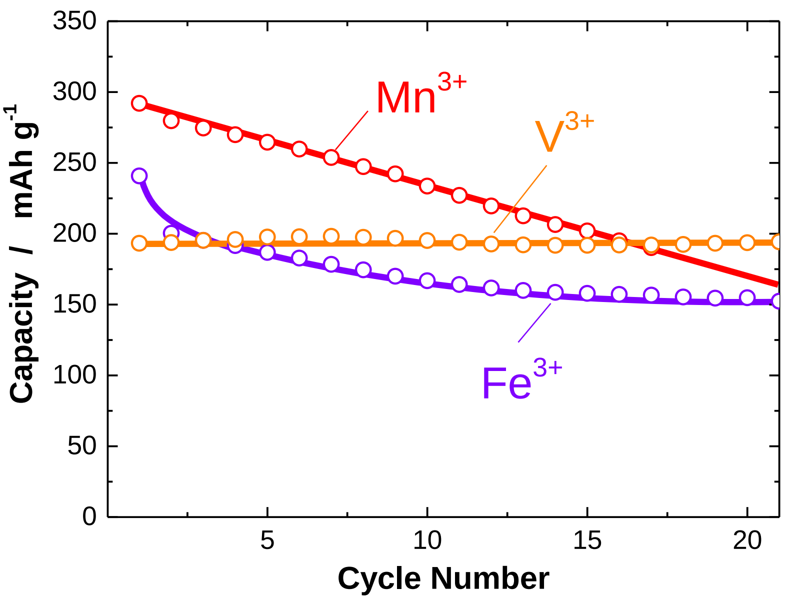

Supplementary Figure 2. Discharge capacity retention of the ball-milled $Li_{1.3\text{-}x}Nb_{0.3}Me_{0.4}O_2$ (Me = Fe^{3+}, Mn^{3+}, and V^{3+}) samples in Li cells. The cells were cycled at a rate of 10 mA g^{-1} at 50 ^{o}C and charge/discharge curves are shown in **Figure 2**.

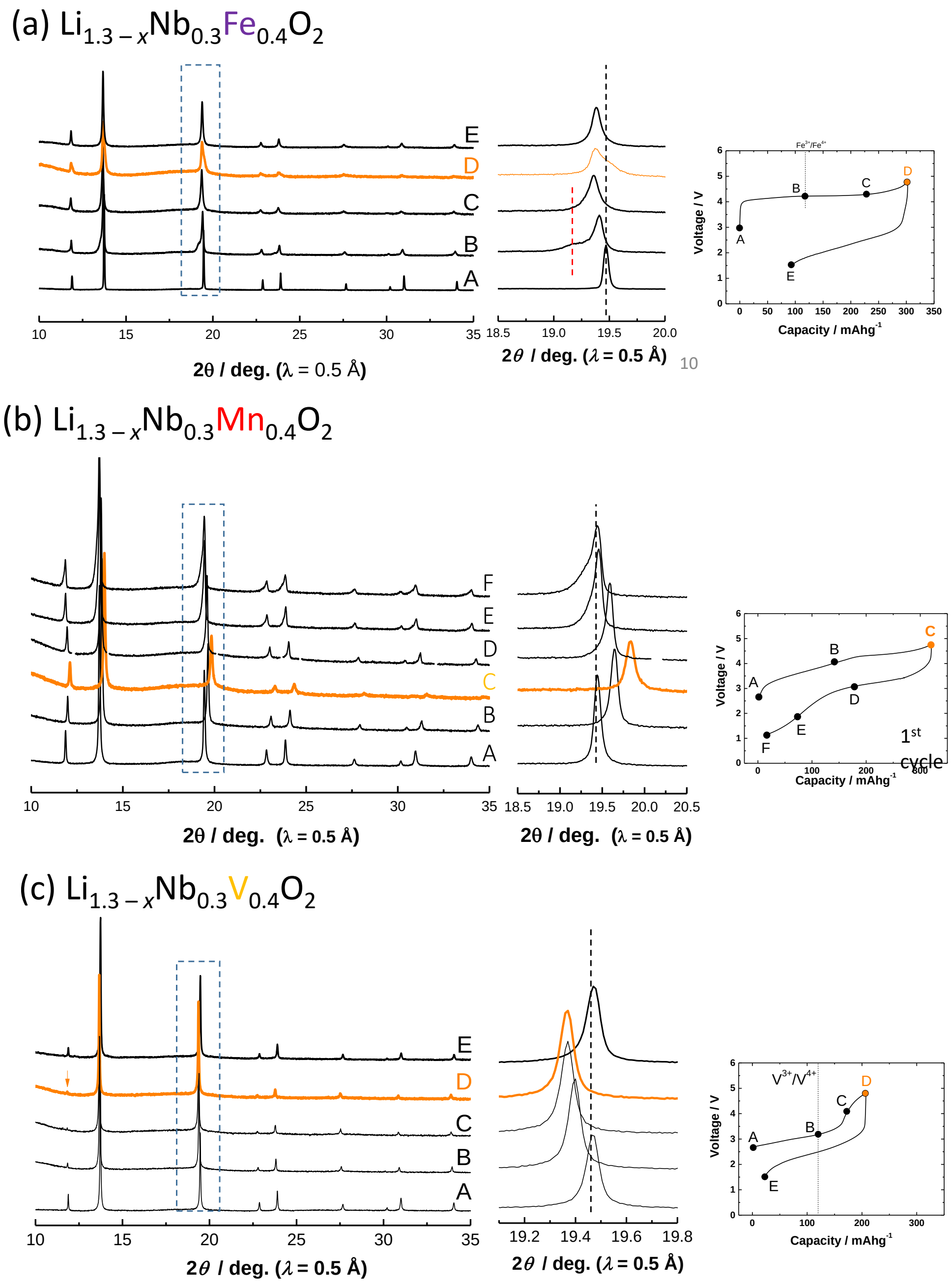

Supplementary Figure 3. *Ex-situ* synchrotron X-ray diffraction patterns (SXRD) of the $Li_{1.3-x}Nb_{0.3}Me_{0.4}O_2$ (Me = Fe^{3+}, Mn^{3+}, and V^{3+}) samples. The data of $Li_{1.3-x}Nb_{0.3}Mn_{0.4}O_2$ have been published in our previous work[1] and shown for the comparison purpose.

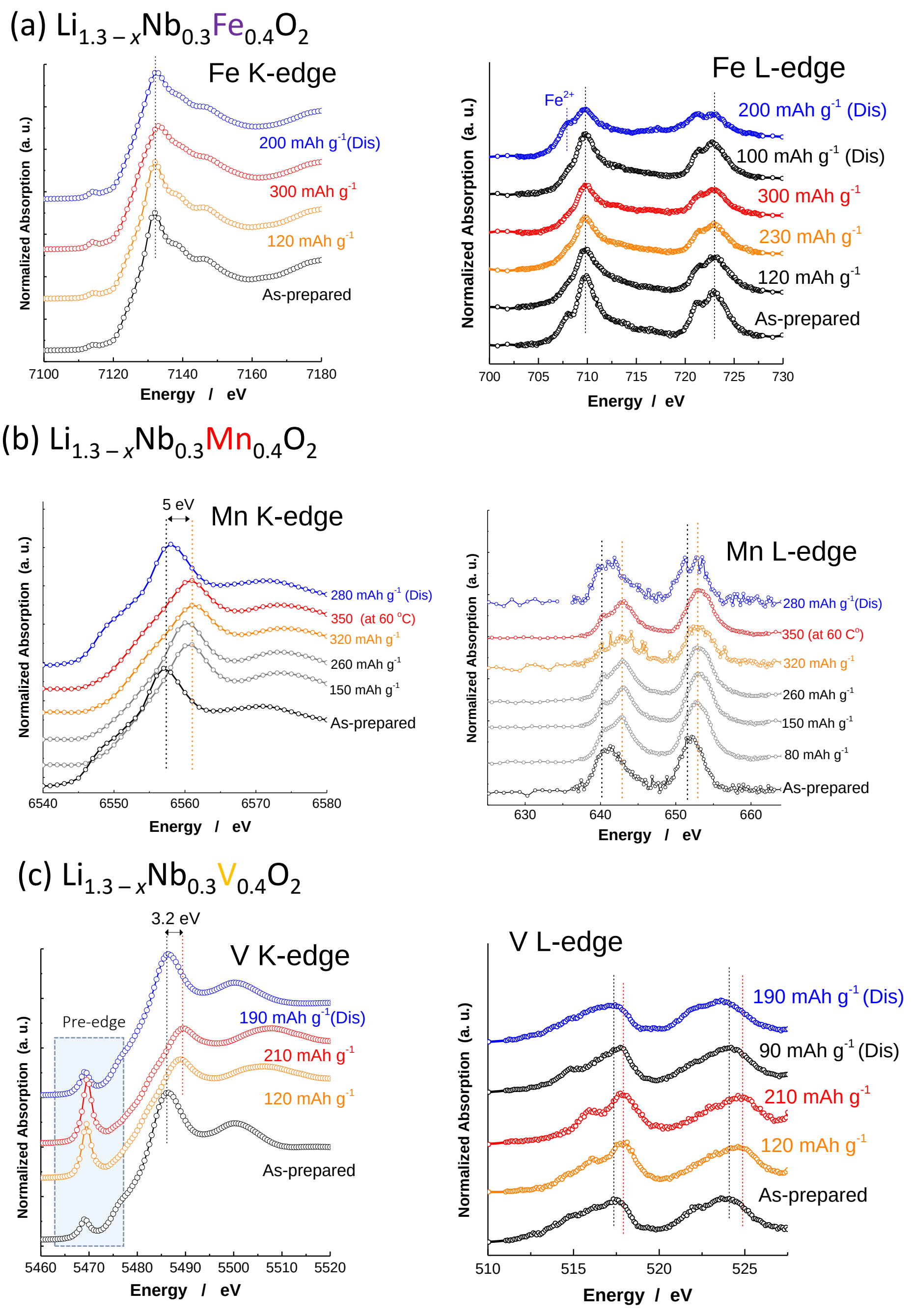

Supplementary Figure 4. Hard/soft X-ray absorption spectroscopy (XAS) spectra of the $Li_{1.3-x}Nb_{0.3}Me_{0.4}O_2$ (Me = Fe^{3+}, Mn^{3+}, and V^{3+}) samples. The K- and L-edge spectra of $Li_{1.3-x}Nb_{0.3}Mn_{0.4}O_2$ and the K-edge spectra of $Li_{1.3-x}Nb_{0.3}V_{0.4}O_2$ have been published in our previous works[1, 2] and shown for the comparison purpose.

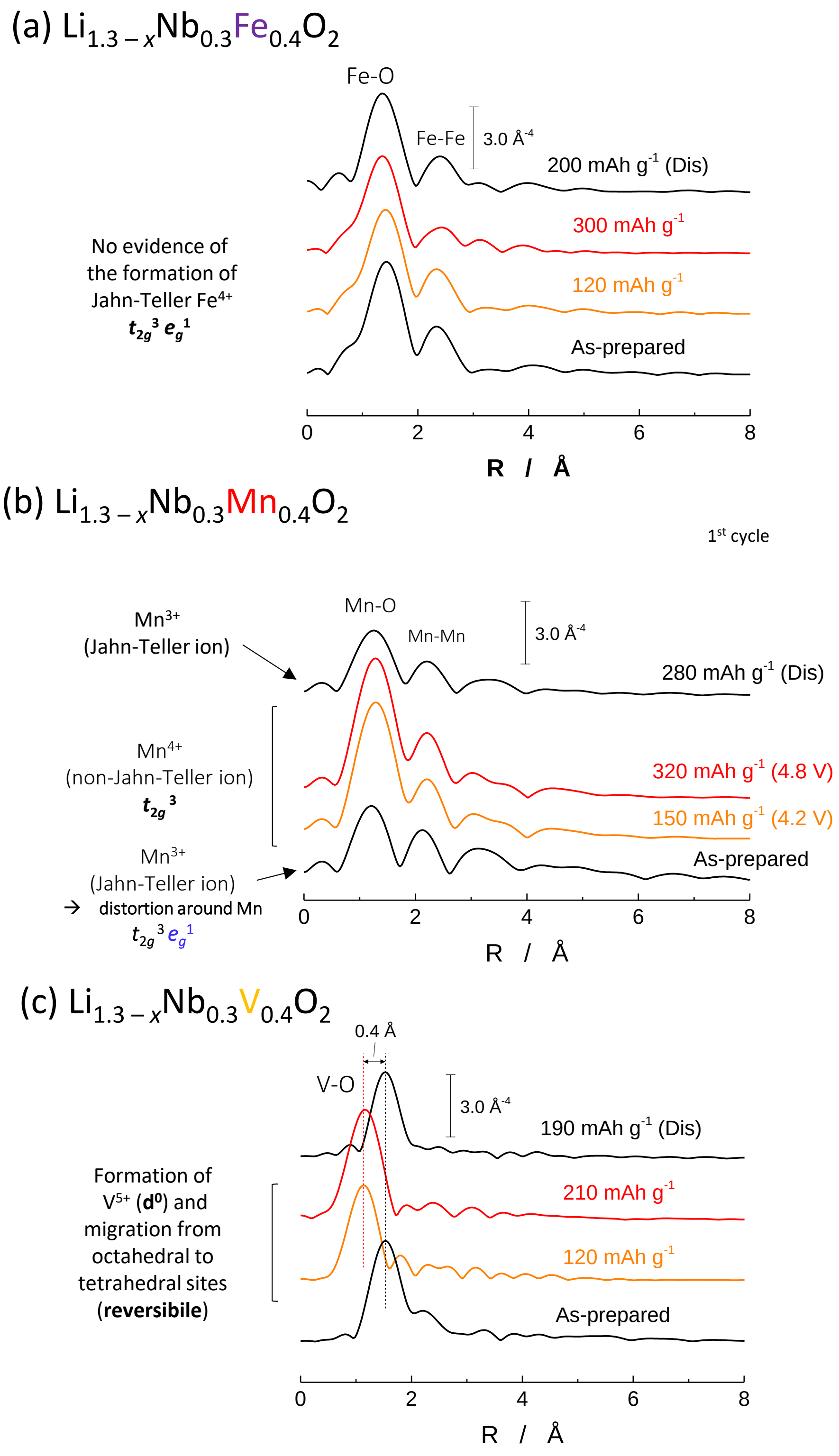

Supplementary Figure 5. Extended X-ray absorption fine structure (EXAFS) of the $Li_{1.3-x}Nb_{0.3}Me_{0.4}O_2$ (Me = Fe^{3+}, Mn^{3+}, and V^{3+}) samples.

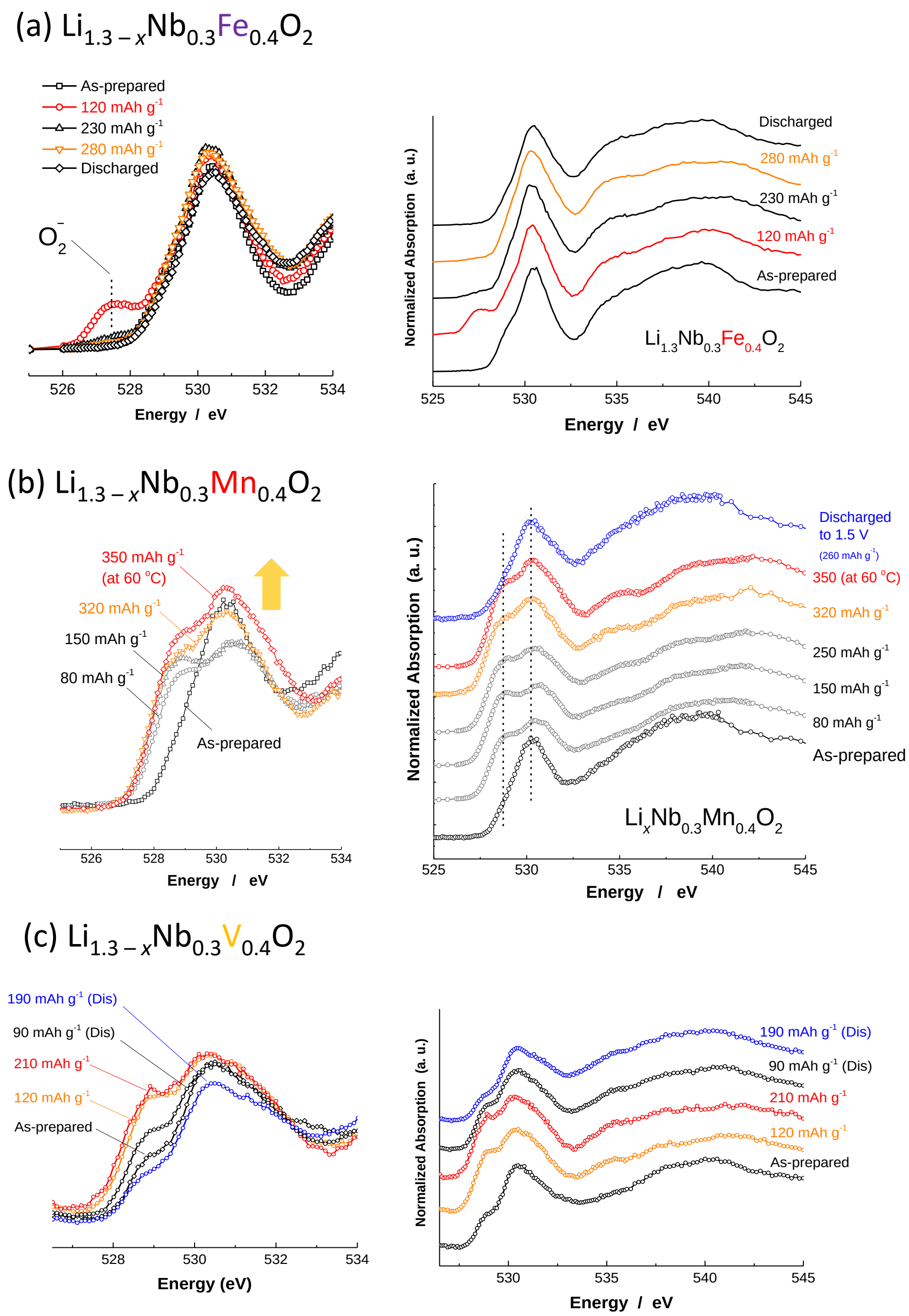

Supplementary Figure 6. Oxygen K-edge XAS spectra of the $Li_{1.3-x}Nb_{0.3}Me_{0.4}O_2$ (Me = Fe^{3+}, Mn^{3+}, and V^{3+}) samples. The data of $Li_{1.3-x}Nb_{0.3}Mn_{0.4}O_2$ have been published in our previous work[1] and shown for the comparison purpose.

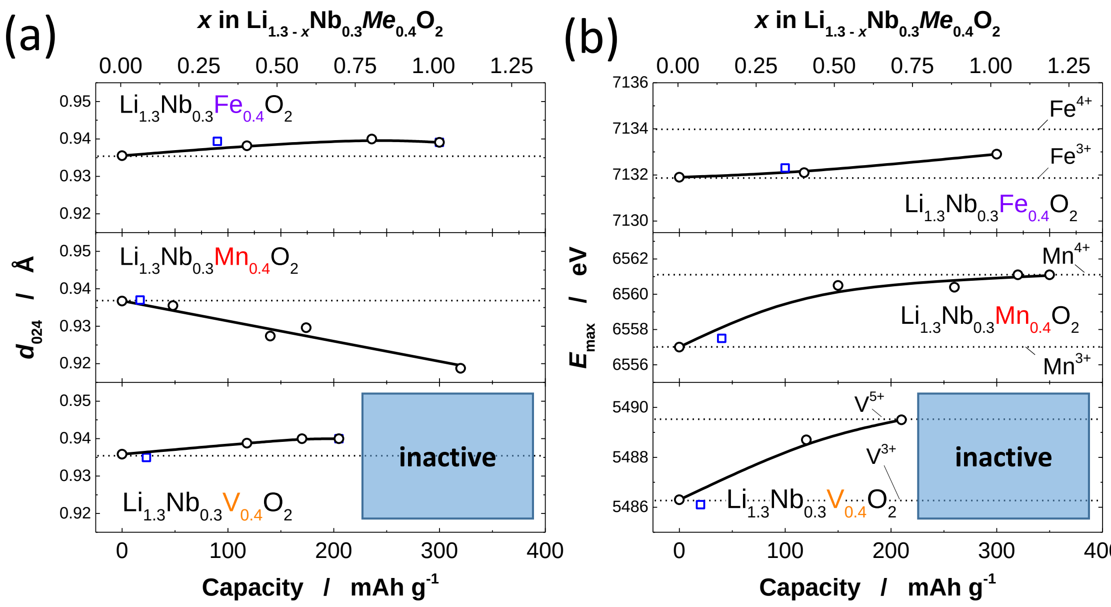

Supplementary Figure 7. Summary of structural and electronical characterization for $Li_{1.3}Nb_{0.3}Me_{0.4}O_2$ (Me = Fe^{3+}, Mn^{3+}, and V^{3+}): (a) changes in interlayer distance observed by SXRD, (b) shift of the energy for the maximum intensity for transition metal K-edge X-ray absorption (XAS) spectra.

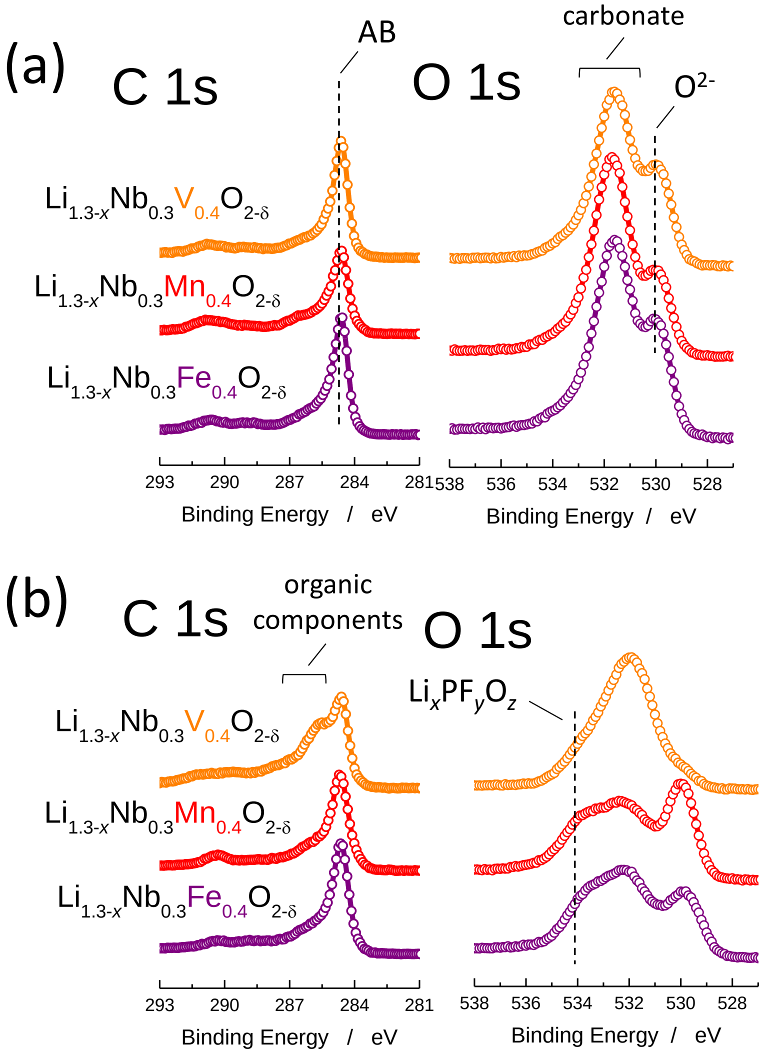

Supplementary Figure 8. X-ray photoelectron spectroscopy (XPS) spectra of the $Li_{1.3-x}Nb_{0.3}Me_{0.4}O_2$ (Me = Fe^{3+}, Mn^{3+}, and V^{3+}) composite electrodes; (a) pristine electrodes and (b) electrodes charged to 4.8 V.

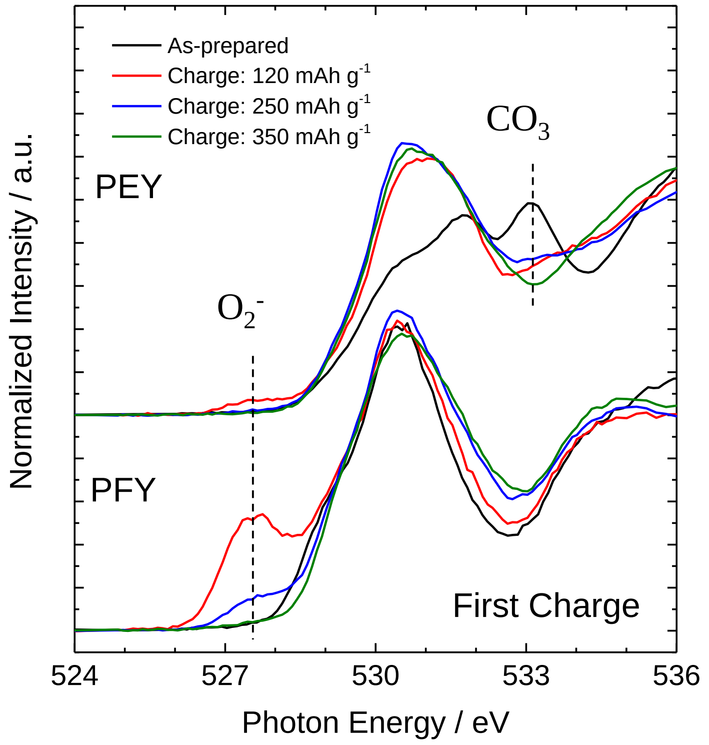

Supplementary Figure 9. Comparison of O K-edge XAS spectra for $Li_{1.3-x}Nb_{0.3}Fe_{0.4}O_2$; partial electron yield (PEY) and partial fluorescence yield (PFY). In general, electronic structures of each element can be obtained from only near the surface of particle for PEY and >100 nm from the surface for PFY. The presence of Li_2CO_3 is found for the as-prepared electrode from the spectra collected by the PEY mode. Since superoxide species is enriched after charge (120 mAh g^{-1}), especially in the spectra of PFY mode, superoxide species is expected to be stabilized in the bulk of particle. The superoxide species near the surface can be readily oxidized to oxygen molecules on further charging.

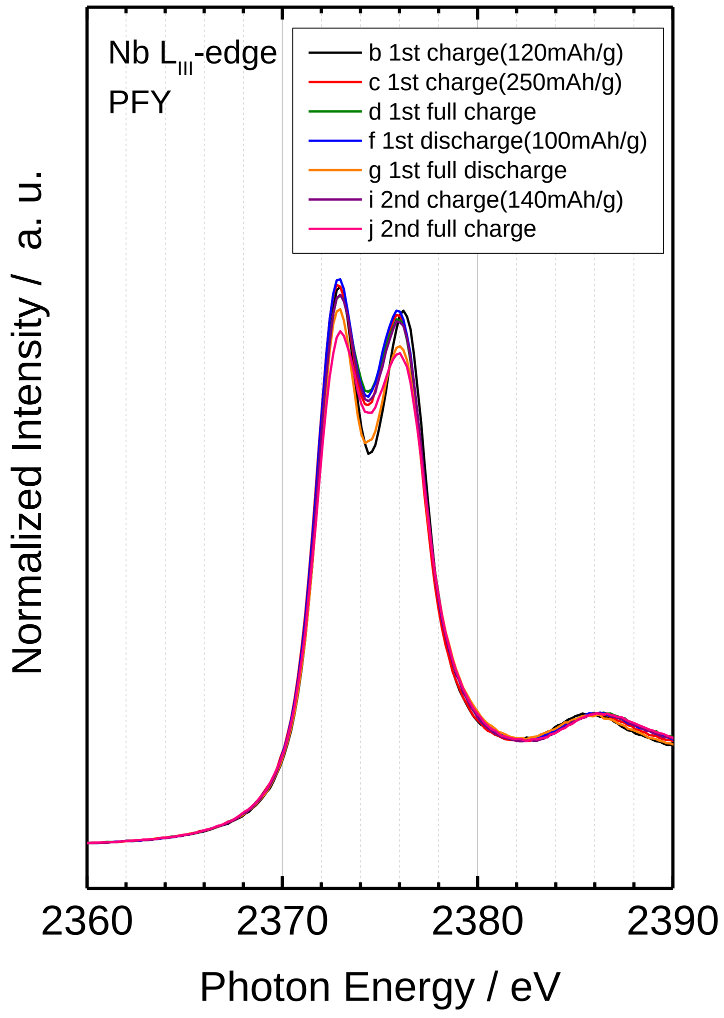

Supplementary Figure 10. Nb L_{III}-edge XAS spectra of $Li_{1.3-x}Nb_{0.3}Fe_{0.4}O_{2-\delta}$ on initial charge/discharge and 2nd charge. Data of the as-prepared sample is shown in **Figure 4D**.

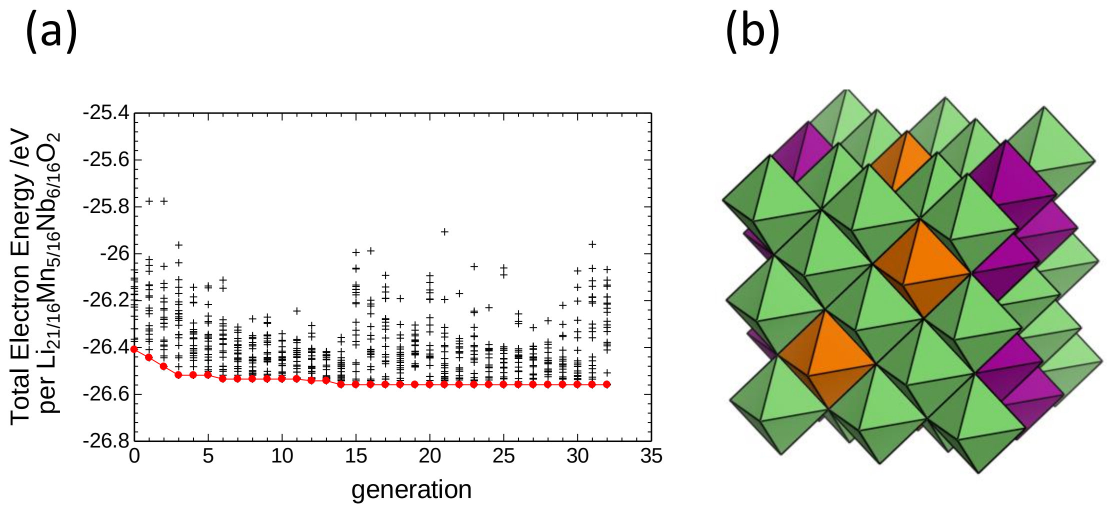

Supplementary Figure 11. (a) Variation of total electron energies for genetic algorithm (GA) driven structures as a function of generation. ($Li_{21/16}Nb_{5/16}Mn_{6/16}O_2$ as an example.) The red symbol indicates the lowest energy of GA generated structures among present and previous generations. (b) Optimized Li/Mn/Nb arrangement for $Li_{21/16}Nb_{5/16}Mn_{6/16}O_2$. Green, purple and orange colored octahedra correspond to LiO_6, MnO_6 and NbO_6, respectively.

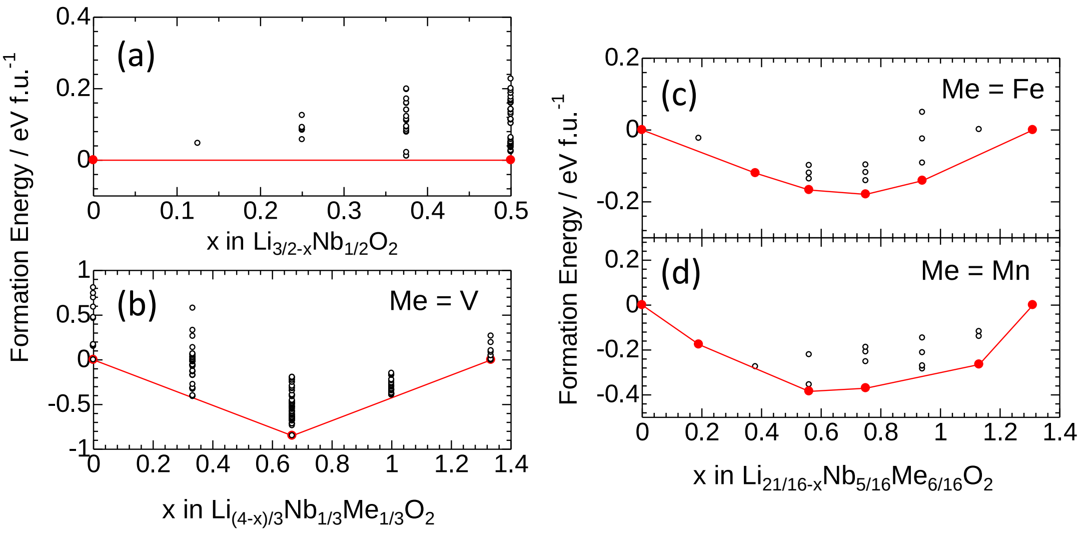

Supplementary Figure 12. (a) Comparison of calculated total energies for various Li/Me/Nb (Me = Fe, Mn, V) cation configurations in rocksalt-type structure with composition of $Li_{4/3}Me_{1/3}Nb_{1/3}O_2$. The lowest energy for each structure is set as zero for the comparison purpose. (b) Calculated formation energies for various Li/vacancy arrangements in rocksalt-type $Li_{4/3-x}Me_{1/3}Nb_{1/3}O_2$. The solid red lines corresponds to the most stable ground states. Blue cross symbols in $Li_{4/3-x}V_{1/3}Nb_{1/3}O_2$ (Me = V) corresponds to the structure where V ions locate at the tetrahedral sites due to migration as indicated in experiments (see text for more details).

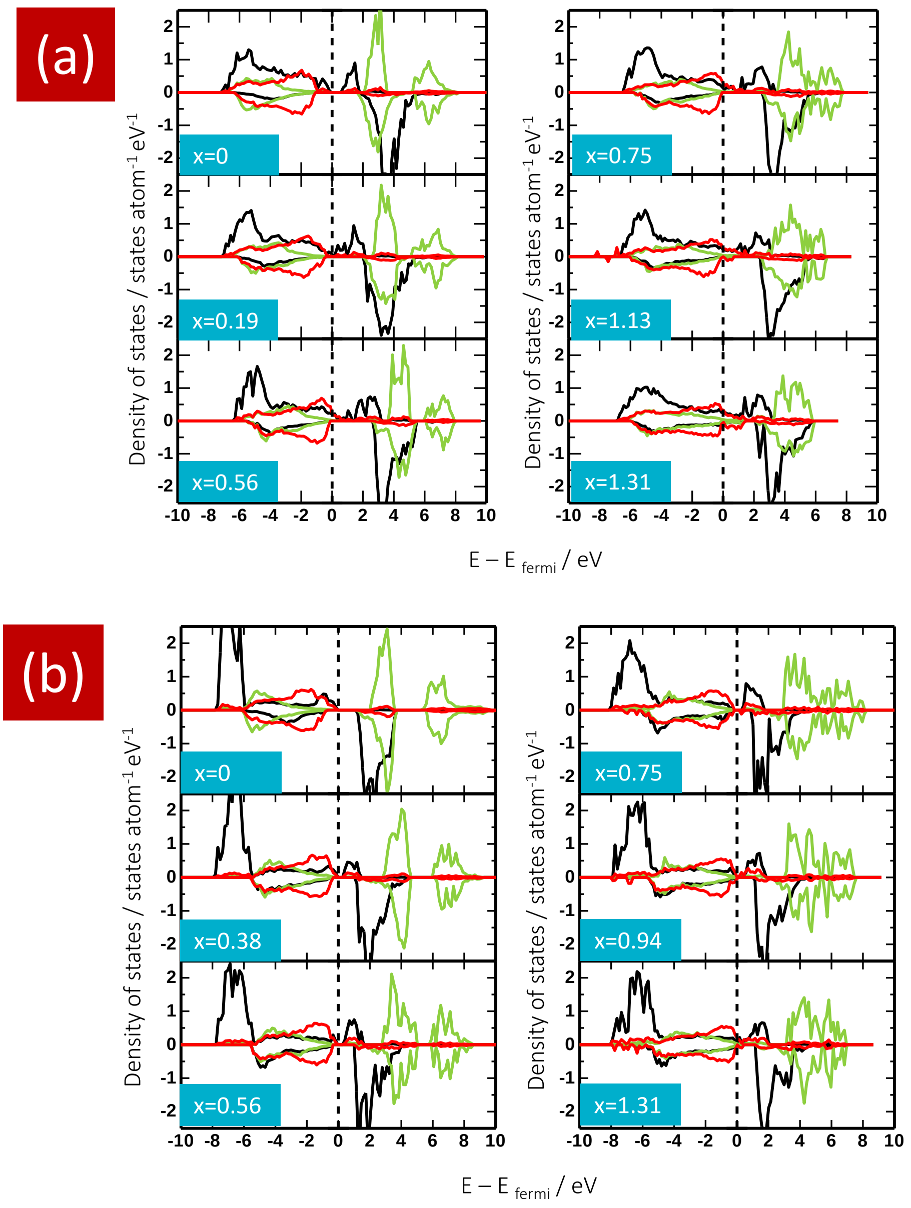

Supplementary Figure 13. Variation of partial density of state (PDOS) around 3d transition metal, niobium, and oxide ions for (a) $Li_{21/16-x}Nb_{5/16}Mn_{6/16}O_2$ and (b) $Li_{21/16-x}Nb_{5/16}Mn_{6/16}O_2$ as a function of composition *x*. Black, green, and red lines indicate PDOS for transition metal, niobium, and oxide ions, respectively.

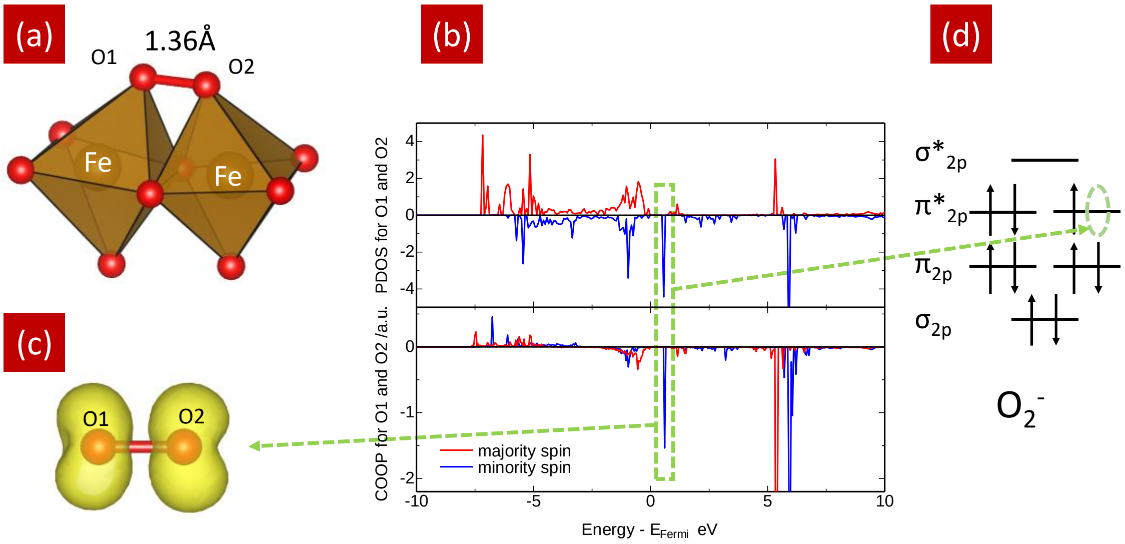

Supplementary Figure 14. (a) The shortest oxygen-oxygen bond at x = 0.75 in $Li_{21/16-x}Nb_{5/16}Mn_{6/16}O_2$ structure. (b) PDOS and COOP profile as a function of energy for the shortest oxygen-oxygen bond. (c) Visualization of charge density distribution for the bottom of the conduction band, consisting of the shortest oxygen-oxygen bond. (d) Schematic figure of electronic configuration for superoxide ion, O_2^{-}.

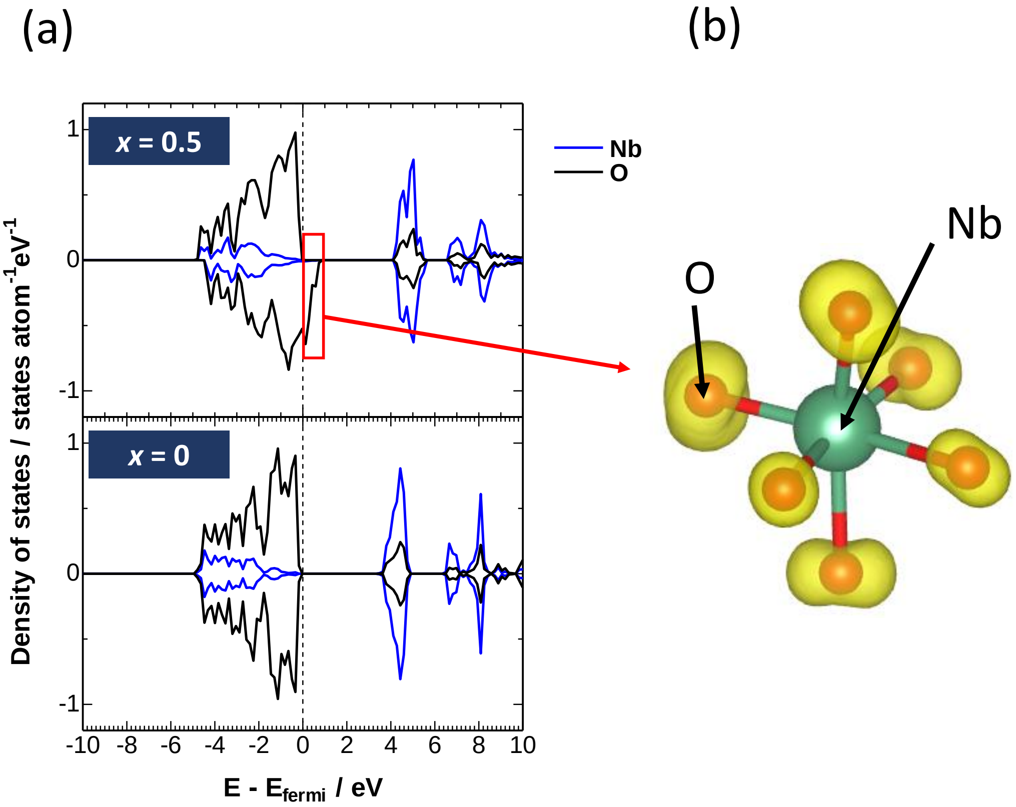

Supplementary Figure 15. (a) PDOS for $Li_{3/2-x}Nb_{1/2}O_2$ ($Li_{3-x}NbO_4$) and (b) visualization of charge density distribution for conduction band bottom of $\square_{0.5}LiNb_{1/2}O_2$ (x = 0.5).

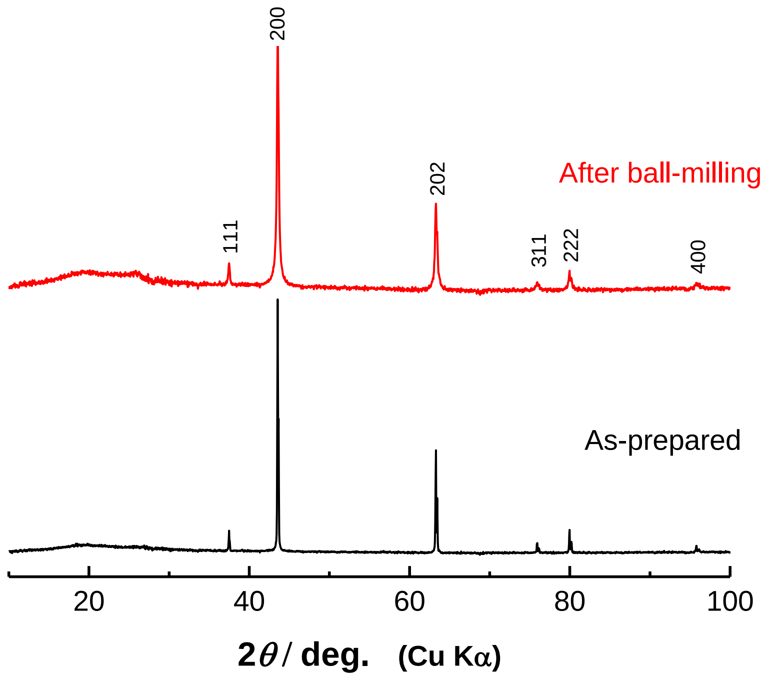

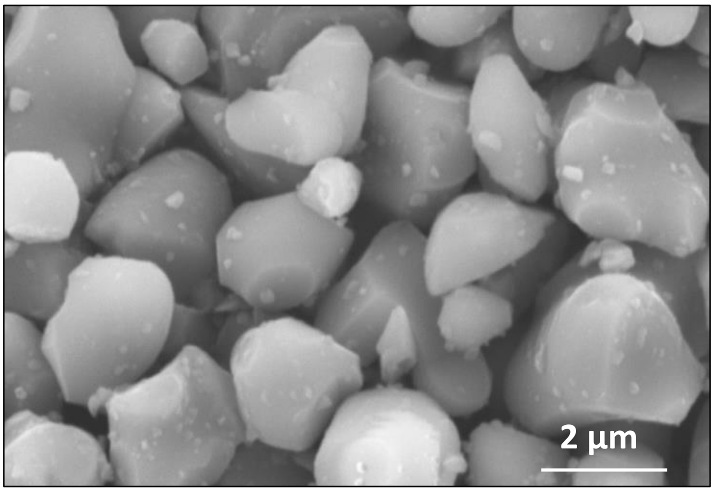

Supplementary Figure 16. (Top) XRD patterns of $Li_{1.2}Ti_{0.4}Mn_{0.4}O_2$; before (as-prepared) and after ball-milling with 10 wt% acetylene black. Diffraction patterns are assigned to the cation-disordered rocksalt-type structure with a lattice parameter of a = 4.150 Å. (Bottom) A SEM image of the as-prepared sample is also shown. The neutron diffraction pattern of the as-prepared sample is also shown in **Figure 5a**.

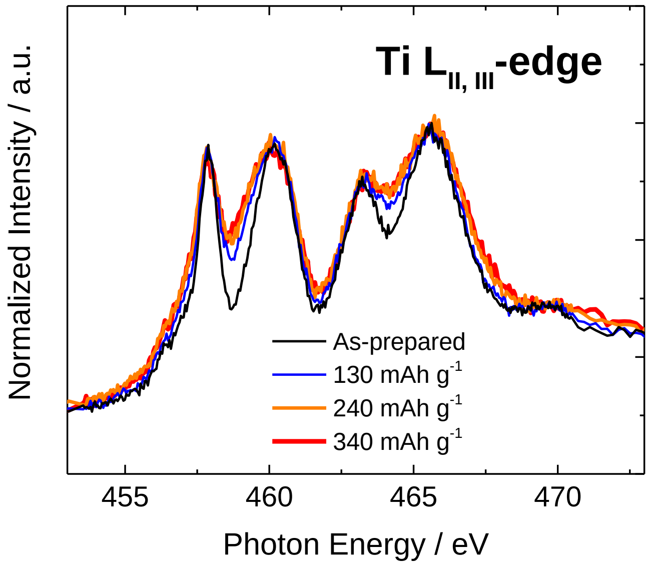

Supplementary Figure 17. Changes in Ti $L_{II, III}$-edge XAS spectra for $Li_{1.2-x}Ti_{0.4}Mn_{0.4}O_2$ on charge in Li cells.

Supplementary Table 1. The shortest bond distances between two oxide ions in Å

Composition *x*	0	0.19	0.38	0.56	0.75	0.94	1.13	1.31
$Li_{21/16-x}Nb_{5/16}Mn_{6/16}O_2$	2.64	2.59	-	2.40	2.25	-	1.31	2.03
$Li_{21/16-x}Nb_{5/16}Fe_{6/16}O_2$	2.70	-	2.43	2.45	1.37	1.30	-	1.30

Supplementary Table 2. Results of Rietveld analysis on the neutron diffraction pattern of $Li_{1.2}Ti_{0.4}Mn_{0.4}O_2$. The sample crystallizes into cation-disordered rocksalt-type structure, and the presence of vacancy at 4*a* site (one percent) is found by Rietveld analysis. The fitting result is shown in **Figure 5a**.

Atom	site	g	x	y	z	B(Å^2)
Li(1)	4a	0.625(3)	0	0	0	0.946(16)^a
Mn(1)	4a	0.198(2)	=x(Li(1))	=y(Li(1))	=z(Li(1))	=B(Li(1))
Ti(1)	4a	1-g(Li(1))-g(Mn(1))-g(Vacancy)	=x(Li(1))	=y(Li(1))	=z(Li(1))	=B(Li(1))
Vacancy	4a	0.012(2)	=x(Li(1))	=y(Li(1))	=z(Li(1))	=B(Li(1))
O(1)	4b	1	1/2	1/2	1/2	1.41(3)^a

Space group $Fm\overline{3}m$ (No. 225), a = 4.156745(4) Å
Histogram A:
$R_{\rm wp} = 5.57$, $R_{\rm p} = 4.31$, $S = R_{\rm wp}/R_{\rm e} = 2.74$, $R_{\rm B} = 3.13$, $R_F = 11.89$.
[a] Equivalent isotropic thermal parameter, B_{eq}.

Supplementary Methods

Synchrotron X-ray diffraction (SXRD): To examine changes in crystal structures of $Li_{1.3-x}Nb_{0.3}Me_{0.4}O_2$ (Me = Fe^{3+}, Mn^{3+}, and V^{3+}), SXRD is applied for the composite electrodes prepared in Li cells at room temperature. Synchrotron X-ray diffraction (SXRD) patterns were collected at beam line BL02B2, SPring-8 in Japan, equipped with a large Debye–Scherrer camera.[3] The $Li_{1.3-x}Nb_{0.3}Me_{0.4}O_2$ (Me = Fe^{3+}, Mn^{3+}, and V^{3+}) samples were electrochemically prepared in Li cells at a rate of 5 or 12 mA g^{-1} at room temperature. The composite electrodes were rinsed with dimethyl carbonate to remove excess electrolyte and then dried at room temperature in the Ar-filled glove box. The composites were separated from the aluminum current collectors, crushed into powders, and then filled into glass capillaries (diameter 0.5 mm). The glass capillaries were sealed using a resin in the glove box to eliminate sample exposure to air. To minimize the effect of X-ray absorption by the samples, the wavelength of incident X-ray beam was set to 0.5 Å using a silicon monochromator, which was calibrated to 0.5001(1) Å with a CeO_2 standard. Structural analysis was carried out using RIETAN-FP.[4]

The data of $Li_{1.3-x}Nb_{0.3}Mn_{0.4}O_2$ has been published in our previous work[1] and are shown for the comparison purpose. Reversible structural changes are observed for the Mn^{3+} and V^{3+} system, as shown in **Supplementary Figure 3**. Shrinkage of the crystal lattice (approximately 6 % by volume) as a function of lithium contents in the structure is observed on charge (lithium extraction) for $Li_{1.3-}$

$_x$Nb$_{0.3}$Mn$_{0.4}$O$_2$. This result is consistent with the fact that transition metals (and oxide ions) are oxidized, and lithium ions are extracted from the crystal lattice.

In contrast, small expansion of the crystal lattice is observed for $Li_{1.3-x}Nb_{0.3}V_{0.4}O_2$. Additionally, the 111 diffraction line on charge disappears, and this diffraction line again appears after discharge. This change suggests the formation of an antifluorite phase (its oxygen packing is the same as rocksalt, ccp, and cations are located at tetrahedral sites) on charge, and this phase transition is the reversible process. These findings suggest the migration of vanadium ions from octahedral to tetrahedral sites on charge, and this result is further supported by XAS and DFT calculation as shown in the later section. Such migration of vanadium ions probably causes small volume expansion (1.3 % by volume) on "*lithium extraction*". Changes in the *d* values are summarized in **Supplementary Figure 7a**.

For $Li_{1.3-x}Nb_{0.3}Fe_{0.4}O_2$, irreversible phase transition is observed in the SXRD patterns. A new phase with a broad profile appears on the early stage of charge (~118 mAh g^{-1}, x = 0.4) with a large unit cell (approximately 4 % larger by volume) compared with the pristine sample. This new phase can correlate with the formation of superoxide-like species as discussed in the later section. Moreover, for the fully discharged sample to 1.5 V, the unit cell volume is larger (1.3 % by volume) than that of the pristine sample. Such increase in the unit cell volume originates from the irreversible phase transition (oxygen loss and densification as observed for the Li_2MnO_3-based electrode

materials) coupled with the reduction of Fe^{3+} to Fe^{2+} on discharge, similar to Li_2MnO_3-based electrode materials.[5-8] This finding is also consistent with the results of XAS as described below.

Hard and soft X-ray absorption spectroscopy (XAS): Hard and soft XAS spectra were collected for the Li_3NbO_4-based samples with different charge/discharge states (**Supplementary Figure 4**) at beam line BL-12C of the Photon Factory Synchrotron Source in Japan. Hard X-ray absorption spectra were collected with a silicon monochromator in a transmission mode. The intensity of incident and transmitted X-ray was measured using an ionization chamber at room temperature. Li_3NbO_4-based composite samples were prepared using the coin cells at a rate of 12 $mA \cdot g^{-1}$. The composite electrodes were rinsed with dimethyl carbonate and sealed in a water-resistant polymer film in the Ar-filled glove box.

No clear change for Fe K-edge X-ray absorption near edge structure (XANES) spectra is found on charge whereas slight shift of K-edge spectra to the lower energy region is noted on discharge. From the extended X-ray absorption fine structure (EXAFS) spectra (**Supplementary Figure 5**), formation of Fe^{4+}, which is known as a Jahn-Teller ion ($t_{2g}^3\ e_g^1$),[9] is also not evidenced. A sharp peak for the first coordination shell of the charged sample indicates high symmetry (O_h or D_{3h}) around iron. Instead, formation of Fe^{2+} after discharge is more pronounced in the Fe L-edge spectrum. This result is consistent with the SXRD observation, *i.e.*, irreversible structural changes and expansion of crystal

lattice after discharge.

The XANES spectra of $Li_{1.3-x}Nb_{0.3}Mn_{0.4}O_2$ has been published in our previous work[1] and is shown for the comparison purpose. For the Mn system, from changes in profiles of XAS spectra, oxidation of Mn^{3+} to Mn^{4+} is found for both Mn K- and L-edges, and these changes are only observed for the slope region (3 – 4 V region). The energy of maximum absorption for the Mn K-edge XANES spectra shifts toward a higher energy region (approximately 5 eV) on charge, but such energy shift is not found on the plateau region at 4.2 V, as summarized in **Supplementary Figure 7b**. Similarly, profiles of Mn L-edge XAS spectra change on the early state of charge (~80 mAh g^{-1}). The Mn L-edge XAS spectra for the pristine and charged samples (~80 mAh g^{-1}) resemble the spectra of Mn^{3+} and Mn^{4+}, respectively.[1] However no change in the spectra is observed in the plateau region at 4.2 V. This observation is further supported from the EXAFS spectra, clear evidence for the presence of Jahn-Teller Mn^{3+} (t_{2g}^3 e_g^1) is noted for the as-prepared and discharged samples. After charge to the plateau region, the presence of Mn^{4+} (t_{2g}^3) as a non-Jahn-Teller ion is found as evidenced from the sharp peak for the 1st coordination shell.

Similar to Mn K-edge and L-edge spectra, obvious changes are found for V K-edge and L-edge spectra. Both spectra for K- and L-edges gradually shift to the higher energy region, and this result is consistent with the change in voltage of the $Li/Li_{1-x}Nb_{0.3}V_{0.4}O_2$ cell. Additionally, a clear change is noted for the pre-edge region of K-edge. Peak intensity of the pre-edge region is intensified

as increase in charge capacity, and this process is a highly reversible process. Such phenomena are often observed for the transition metals with d^0 configuration, which are located at tetrahedral sites.[10] This result suggests vanadium migration from octahedral to tetrahedral sites, and is consistent with the short interatomic distance for the first coordination shell observed for the EXAFS spectra. The V-O bond length for the first coordination shell is reduced by 0.4 Å after charge to 4.8 V. Together with these results and observation of SXRD (changes in intensity of the diffraction lines), it is concluded that vanadium ions are oxidized from the trivalent to pentavalent state, and migrate to the tetrahedral sites. Moreover, this process is the highly reversible process, and vanadium ions are reversibly reduced to trivalent state on discharge and migrate back into octahedral sites, leading to excellent capacity retention based on the two-electron redox process.

Changes in oxygen K-edge spectra: Oxygen K-edge XAS measurement is the effective characterization technique to examine changes in electronic structures of oxide ions.[1, 11] **Supplementary Figure 6** displays changes in O K-edge XAS spectra for three different Li_3NbO_4-based samples on charge/discharge processes. Since oxide ions form chemical bonds with transition metals, oxidation of transition metal ions also affects the profile of O K-edge XAS spectra. For the Mn system, a pre-edge peak centered at 530.5 eV for the as-prepared sample separates into two peaks at 529 and 530.5 eV (the sample charged to 80 mAh g^{-1}). Such change is associated with the

oxidation of Mn^{3+} to Mn^{4+} at the slope region (3 to 4 V region). On the further oxidation (150 – 350 mAh g^{-1}), clear and systematic changes in O K-edge XAS spectra are observed, and clear increase in the intensity for the pre-edge region is observed (**Supplementary Figure 6**) as reported in our previous work.[1] The data of $Li_{1.3-x}Nb_{0.3}Mn_{0.4}O_2$ has been published in our previous work[1] and is shown for the comparison purpose. In the plateau region, no change is evidenced for Mn L- and K-edges XAS spectra. Similarly, the profiles of Nb spectra were not affected by charge/discharge.[1] Systematic studies on the O K-edge XAS spectra for Li_2O_2, Li_2CO_3, and Li_2O have been conducted and these chemical species can be distinguished from the appearance of specific peaks.[12] Li_2O_2 shows (and other peroxide compounds, like zinc peroxide, ZnO_2) shows strong peak at 530 eV. Therefore, these results suggest that oxide ions are oxidized and the formation of peroxide-like species or holes occurs at the plateau region. However, it is noted that the formal oxidation state for the fully charged state (1.2 moles of Li extraction) is estimated to be $\square_{1.2}Li_{0.1}Nb_{0.3}^{5+}Mn_{0.4}^{4+}O_2^{1.6-}$ when all excess charges are assumed to be compensated by the oxidation of oxide ions. The formal oxidation state of oxygen is still far from “*pure*” peroxide ions, and therefore further increase in the capacity is anticipated.

For the Fe system, although a large initial charge capacity is observed, but clear changes in voltage profile is noted from the discharge process (**Figure 2**). Similar to electrochemistry for the Fe system, a clear and interesting difference is found for the O K-edge XAS spectra. On the fully charged state, increase in the intensity for the pre-edge region at 530 eV is not found, indicating that

the formation of peroxide-like species or holes is not eliminated for the Fe system. Instead, a new peak centered at 526.5 eV appears in the early state of charge (120 mAh g^{-1}), and this peak is assigned into superoxide species, O_2^{-}.[13] This peak disappears further charge, suggesting that superoxide species are electrochemically oxidized and decomposed to oxygen molecules on charge. This fact also suggests that oxygen loss and the densification of the crystal lattice for the Fe system, similar to Li_2MnO_3-based system (however this process is more pronounced for the Fe-Nb system, as shown in **Figure 2**). This finding is also consistent with the observation by SXRD, XAS, and electrochemical data. Continuous oxygen loss occurs in the Fe system and peroxide-like species or holes is not stabilized in this system.

O K-edge XAS spectra for the V system are also shown in **Supplementary Figure 6c**. As described above, V^{3+} ions are oxidized to V^{5+} on charge and small V^{5+} ions migrate to tetrahedral sites, at which V^{5+} ions are energetically stabilized. These processes inevitably result in the changes in the profiles of O K-edge XAS spectra. Nevertheless, increase in the intensity at 529 eV is pronounced and a clear change at 530 eV is not observed. Together with these results coupled with electrochemical data, it is concluded that oxide ions are not oxidized in the V system. This fact can be explained as follows: Electrochemical redox potential of V^{3+}/V^{5+} in the framework of oxide ions is lower than that of the oxidation of oxide ions (approximately 4 V vs. Li estimated from the Fe system). This fact indicates that electrochemically active vanadium ions are fully oxidized to V^{5+} before the

oxidation of oxide ions is activated. At the same time, electronic conductivity after the oxidation of V^{3+} to V^{5+} is inevitably reduced because of the absence of *d* electrons in a conduction band. As shown in **Figure 1**, as-prepared black colored V system (with V^{3+}) shows high electronic conductivity whereas V^{5+} without *d* electrons shows intrinsically low electronic conductivity. It is reasonably expected that the fully charged sample, $\square_{0.8}Li_{0.4}Nb_{0.3}^{5+}V_{0.4}^{5+}O_2$, shows insufficient electronic conductivity to activate the oxidation of oxide ions, similar to Li_3NbO_4.

Surface analysis by X-ray photoelectron spectroscopy (XPS): XPS is a powerful technique to examine a surface state of composite electrodes. We have reported that oxygen gas released on initial charge of the Li_2MnO_3-based electrode materials results in the formation of surface deposits on discharge.[7] Oxygen gas released on charge is electrochemically reduced on discharge, like Li-Air batteries. Electrochemical reduction of oxygen molecules results in the formation of superoxide, which further reacts with carbonate-based solvents, leading to the accumulation of surface deposits. Therefore, the amount of oxygen gas released on charge is indirectly estimated by XPS as reported in the literature.[7, 14, 15]

XPS spectra of the composite electrodes of $Li_{1.3}Nb_{0.3}Me_{0.4}O_2$ (Me = V^{3+}, Mn^{3+}, and Fe^{3+}) have been collected with different conditions; pristine, charged to 4.8 V (**Supplementary Figure 8**), and discharged to 1.5 V (after charge to 4.8 V) in **Figure 4E**. For the pristine electrodes, O 1s XPS

spectra mainly consist of two components; oxide ions at 529.8 eV and surface impurities on the oxide particles, *e.g.*, lithium carbonate, at *ca*. 532 eV. Additionally, the presence of conjugated carbon originating from acetylene black in the composite electrodes is found as a major component in C 1s spectra at 284.6 eV. Components of PVdF binder, $–CH_2–$ and $–CF_2–$ are also found at 286.2 and 290.8 eV, respectively.[16]

After charge to 4.8 V, some differences are observed for both O 1s and C 1s XPS spectra. For the C 1s XPS spectra, the peak intensity of acetylene black is relatively wakened, and increase in the intensity assigned to organic components (*e.g.*, –C–O–, –C=O *etc*. presumably derived from the decomposition of carbonate-based electrolytes) at 286 – 287 eV is observed. Decomposition of electrolyte on charge is also supported by the appearance of phosphate species, $Li_xPF_yO_z$,[16] in the O 1s XPS spectra. In addition, the peak intensity of oxide ions is intensified after charge to 4.8 V for the Fe and Mn systems, but not for the V system. Such trend for the Fe and Mn systems, increase in the intensity of oxide ions and decrease in the intensity of carbonate impurities on the surface is consistent with the literature.[7] Since lithium carbonate is electrochemically oxidized and decomposed on charge to 4.8 V (above 4.0 V vs. Li),[7] the intensity from the carbonate species is expected to be reduced. For the vanadium sample, such phenomena was not observed. This result also supports the fact that solid-state redox reaction of oxide ions was not activated in the V system. Carbonate impurities must exist on the surface of oxide particles, and not on the acetylene black.

Therefore, similar to the activation of oxide ions, decomposition of carbonate impurities is significantly influenced by the electronic conductivity of samples. Since the V system loses the electrical conductivity for the fully charged state, as discussed above, the decomposition of carbonate impurities, above 4 V, is not observed.

After discharge to 1.5 V, clear changes are noted for three different electrodes as shown in **Figure 4E**. No change is observed for the V system before and after discharge to 1.5 V, indicating that the possibility of oxygen loss on charge is eliminated in this system. For the Fe system, a clear evidence of the accumulation of surface deposits is found as expected from the formation of the superoxide-like species. The intensity of the peak originating from acetylene black is significantly weakened for the C 1s XPS spectrum. Instead, carbonate species is enriched in the O 1s XPS spectrum. Similar trend has been reported in the literature for the Li_2MnO_3-based electrode materials.[7, 14, 17] This observation also supports that the significant oxygen loss occurs on charge for the Fe system. For the Mn system, the peak intensity of carbonate species is slightly increased after discharge to 1.5 V compared with the electrode charged to 4.8 V. This result suggests that oxygen loss is not completely suppressed in the Mn system, but the formation of carbonate species is clearly reduced compared with the conventional Li_2MnO_3-based electrode materials, and thus oxygen loss seems to be comparable to that of Li_2RuO_3 system.[18]

Density functional theory calculations: First-principles calculations based on density functional theory (DFT) for Li_3NbO_4–$LiMeO_2$ (Me = Fe, Mn, and V) system were performed using Vienna ab initio simulation package (VASP)[18, 19] with modified Perdew-Burke-Ernzerhof generalized gradient approximation (PBEsol-GGA)[19, 20] and with the projector-augmented wave (PAW) method.[21] Besides, the on-site Coulomb correction (GGA + *U*) was included for localized electronic states, and *U* value was chosen to be 4.0, 3.9, 3.1, and 1.5 eV for *d* states of Fe, Mn, V, and Nb, respectively, according to the literature.[22] The molar ratio of Me and Nb was set as 1:1 for Me = V, *i.e.* $Li_{4/3-x}Me_{1/3}Nb_{1/3}O_2$, and 6:5 for Me = Mn and Fe, *i.e.* $Li_{21/16-x}Nb_{5/16}Me_{6/16}O_2$, in the present DFT computations for convenience sake, unless specially mentioned hereinafter. (The experimental molar ratio of Me:Nb is 4:3.)

The cation configuration of undoped compound, Li_3NbO_4, was referred to the reported structure,[23] in which Li and Nb ions forms ordered arrangement. (s.g. *I*23, cubic). On the other hand, we used genetic algorithm (GA) approach to explore reasonable Li/Me/Nb arrangement for $Li_{21/16}Nb_{5/16}Me_{6/16}O_2$ (Me = Mn, Fe). We also used genetic algorithm (GA) approach to explore reasonable cation configuration efficiently. In detail, 2 x 2 x 2 conventional rocksalt structure, i.e. $Li_{21}Nb_5Me_6O_{32}$, is used in the present GA procedure, and cation arrangement is regarded as ternary string, consisting of three labels, 0 (Li), 1 (Me) and 2 (Nb), and each cationic site is assigned to the specific index of the string. Therefore, the number of labels “0”, “1”, and “2” corresponds to 21, 6,

and 5, respectively. We prepared at first 20 configurations (ternary strings), where Li, Me, and Nb are arranged randomly at the cation sites (1st generation), and computed total electron energies. Twelve low energy configurations among 20 candidates (60 %) are selected as survivors and their structural features are succeeded to the next generation by following four options. (1) three best structures are succeeded as they are, (2) 8 new structures are created by two-point crossover technique, (3) 8 are half uniform crossover technique, and (4) 4 are mutation techniques. The generated structure was selected not to be consistent with previously calculated configuration. The details of genetic algorithm are described elsewhere.[24, 25] By repeating this procedure, the lowest energy configuration is determined heuristically. Note that we obtained total energies during GA procedure by reducing energy cutoff and number of *k* points in the reciprocal cell to 360 eV and unity (Γ point sampling), respectively, in order to speed up each calculation, since the total energies around ~1000 configurations are needed to evaluate. After GA cycles, obtained lowest-energy configurations are recalculated with sufficient size of energy cutoff (500 eV) and *k* point mesh ($2 \times 2 \times 2$ *k*-point meshes). **Supplementary Figure 11** presents a typical example of GA cation configuration optimization and GA driven structures.

On the other hand, some of DFT computations require relatively large computational time for Me = V during the GA approach due to strong tetrahedral site preference and migration behavior of V ions during structural relaxation. Instead, the cation configuration of $Li_{4/3}V_{1/3}Nb_{1/3}O_2$ with rock-

salt structure is determined by calculating total electron energies for symmetrically distinct configurations with 30 Li/V/Nb arrangements using ATAT software package.[26-30]

Next, electrochemical delithium process for Li_3NbO_4, $Li_{4/3}V_{1/3}Nb_{1/3}O_2$, and $Li_{21/16}Nb_{5/16}Me_{6/16}O_2$ (Me = Mn, Fe) with the lowest energy cation configurations are computed. In order to determine Li/vacancy arrangement, GA approach is adopted for $Li_{21/16-x}Nb_{5/16}Me_{6/16}O_2$ (Me = Mn, Fe), whereas survey of symmetrically distinct configurations for $Li_{3-x}NbO_4$ and $Li_{4/3-x}V_{1/3}Nb_{1/3}O_2$ composition to determine Li/vacancy arrangement. Phase stability of delithiated samples are evaluated by plotting formation energies, ΔE_f. For example, ΔE_f for $Li_{3-x}NbO_4$ ($0 \leq x \leq 1$) can be defined as

$$\Delta E_f\,(Li_{3-x}NbO_4) = E_0(Li_{3-x}NbO_4) - (1 - x)\,E_0(Li_3NbO_4) - x\,E_0(Li_2NbO_4) \qquad \text{(s1)}$$

where E_0(X) corresponds to total electron energy of composition X.[31] **Supplementary Figure 12** displays calculated formation energies for various Li/vacancy configurations. The solid red lines connect the most stable ground states which were found in this computations, and we used to evaluate the voltage as shown in **Figure 3 (A-D)** in the main text. Note that the cell voltage, V, was computed according to the literature[32] as below;

$$V = -\{E_0(Li_{4/3-x}MeNbO_2) - E_0(Li_{4/3-x-z}MeNbO_2) - zE_0(Li)\}zF, \qquad \text{(s2)}$$

where z and F stand for the charge in electrons transported by corresponding Li^+ exchange reaction and the Faraday constant, respectively. As indicated in the XANES spectra (**Supplementary Figure**

4), vanadium ions seem to migrate and occupy at the tetrahedral vacancy site of the cubic close packed (ccp) oxygen array. It is also calculated the configurations which allows tetrahedral site occupancies of V ions for $Li_{4/3-x}V_{1/3}Nb_{1/3}O_2$ as represented by blue cross symbols in **Supplementary Figure 10b**. Indeed, several configurations including vanadium ions sitting at tetrahedral sites form the ground states. Examples are x = 2/3 and 4/3, in which 170 and 70 meV per $Li_{4/3-x}V_{1/3}Nb_{1/3}O_2$ lower than the structures that contain vanadium ions occupying only at octahedral sites whereas no tetrahedral occupancy is indicated for the pristine composition of $Li_{4/3}V_{1/3}Nb_{1/3}O_2$. These results agree well with the experimental observations (**Supplementary Figures 3-5**).

DFT results suggests that topotactic reaction occurs for the Fe system as shown in **Figure 3a**. However, structural instability and oxygen loss are experimentally proposed. Phase stability of $\square_{2/3}Li_{2/3}Fe_{1/3}Nb_{1/3}O_2$ is, therefore, computationally evaluated. Thus, the decomposition reaction is assumed as shown below and the reaction energy was calculated based on this equation (s3).

$$\square_{2/3}Li_{2/3}Fe_{1/3}Nb_{1/3}O_2 \rightarrow 1/3\ LiFeO_2 + 1/3\ LiNbO_3 + 1/6\ O_2 \qquad (s3)$$

For comparison purpose, the reaction energy for the decomposition process in $\square_{2/3}Li_{2/3}Mn_{1/3}Nb_{1/3}O_2$ is also evaluated by the corresponding equation (s4).

$$\square_{2/3}Li_{2/3}Mn_{1/3}Nb_{1/3}O_2 \rightarrow 1/3\ LiMnO_2 + 1/3\ LiNbO_3 + 1/6\ O_2 \qquad (s4)$$

Calculated reaction energy for decomposition of the half delithiated samples, Me = Fe and Mn, are –280 and +350 meV / $\square_{2/3}Li_{2/3}Me_{1/3}Nb_{1/3}O_2$, respectively. Negative reaction energy for Fe indicates

the decomposition reaction proceeds thermodynamically, and vice versa. This result agrees with the experimental indication that electrochemical delithiation for $Li_{1.3-x}Nb_{0.3}Fe_{0.4}O_2$ lead to the decomposition with oxygen loss whereas $Li_{1.3-x}Nb_{0.3}Mn_{0.4}O_2$ compounds are stable during electrochemical charge and discharge. The total energy of $Li_{2/3}Me_{1/3}Nb_{1/3}O_2$ (Me = Mn, Fe) is also calculated along with the procedure of $Li_{2/3}V_{1/3}Nb_{1/3}O_2$. Note that total energy of an oxygen molecule is corrected along with the literature [33] in order to take into account discrepancy related to factors such as over-binding of the GGA.[33-35]

COOP analysis and the formation of superoxide: Table S1 displays calculated the shortest bond distances between neighboring oxide ions during electrochemical delithiation for $Li_{21/16-x}Me_{6/16}Nb_{5/16}O_2$ (M = Mn and Fe). The O-O bond distance is gradually shortened due to oxidation of transition metals (Me^{3+} → Me^{4+}) and oxide ions ($O2^{-}$ → O^{-}) at the early stage of delithiation. Further Li removal from x = 0.56 to 0.75 in the Fe system makes the shortest O-O bond distance decreased largely from ~ 2.4 to ~ 1.35 Å. **Supplementary Figure 14(a)** displays configuration around the shortest O-O bond at x = 0.75 of the Fe system. (Two oxygen atoms are labeled as O1 and O2.) **Panel (b)** shows PDOS and crystal orbital overlap population (COOP)[36] for the O1 and O2 atoms. The COOPs were computed by lobster program.[37-39] Note that positive and negative COOP indicate bonding and antibonding orbital, respectively. Around the top of valence band (from > – 2.5

eV to Fermi level), both up (majority) and down (minority) spin electrons occupies antibonding orbital, whereas down spin states appears at the bottom of conduction band (~0.5 eV versus Fermi level) as antibonding orbital. Partial charge density distribution at the corresponding energy range is visualized as shown in **panel (c)**. The figure clearly indicates the formation of antibonding π^* type orbital, since O 2p-like robes are perpendicular to the shortest O-O bonds. Therefore, the shortest O-O bonds are ascribed to the superoxide ion, O^{2-}, according to its electronic configuration (**panel (d)**). Note that all π^* orbital are filled with electrons for the case of peroxide ion, O_2^{2-}. Similar behavior is also observed at the later stage of delithiation, $x = 1.13$, for the Mn system. This fact indicates that the formation of superoxide ion is easier for the Fe system than that of the Mn system, which is consistent with experimental findings.

Supplementary References

1. Yabuuchi, N. et al. High-capacity electrode materials for rechargeable lithium batteries: Li_3NbO_4-based system with cation-disordered rocksalt structure. *Proc. Natl. Acad. Sci.* **112**, 7650-7655 (2015).
2. Yabuuchi, N. et al. Synthesis and electrochemical properties of $Li_{1.3}Nb_{0.3}V_{0.4}O_2$ as a positive electrode material for rechargeable lithium batteries. *Chem. Commun.* **52**, 2051-2054 (2016).
3. Nishibori, E. et al. The large Debye-Scherrer camera installed at SPring-8 BL02B2 for charge density studies. *Nuclear Instruments & Methods in Physics Research Section a-Accelerators Spectrometers Detectors and Associated Equipment* **467**, 1045-1048 (2001).
4. Izumi, F. & Momma, K. Three-Dimensional Visualization in Powder Diffraction. *Solid State Phenom.* **130**, 15-20 (2007).
5. Armstrong, A.R. et al. Demonstrating oxygen loss and associated structural reorganization in the lithium battery cathode $Li[Ni_{0.2}Li_{0.2}Mn_{0.6}]O_2$. *J. Am. Chem. Soc.* **128**, 8694-8698 (2006).

6. Jiang, M., Key, B., Meng, Y.S. & Grey, C.P. Electrochemical and Structural Study of the Layered, "Li-Excess" Lithium-Ion Battery Electrode Material $Li[Li_{1/9}Ni_{1/3}Mn_{5/9}]O_2$. *Chem. Mater.* **21**, 2733-2745 (2009).
7. Yabuuchi, N., Yoshii, K., Myung, S.-T., Nakai, I. & Komaba, S. Detailed Studies of a High-Capacity Electrode Material for Rechargeable Batteries, $Li_2MnO_3-LiCo_{1/3}Ni_{1/3}Mn_{1/3}O_2$. *J. Am. Chem. Soc.* **133**, 4404-4419 (2011).
8. Koga, H. et al. $Li_{1.20}Mn_{0.54}Co_{0.13}Ni_{0.13}O_2$ with Different Particle Sizes as Attractive Positive Electrode Materials for Lithium-Ion Batteries: Insights into Their Structure. *J. Phys. Chem. B* **116**, 13497-13506 (2012).
9. Yabuuchi, N. et al. P2-type $Na_x[Fe_{1/2}Mn_{1/2}]O_2$ made from earth-abundant elements for rechargeable Na batteries. *Nat. Mater.* **11**, 512-517 (2012).
10. Yamamoto, T. Assignment of pre-edge peaks in K-edge x-ray absorption spectra of 3d transition metal compounds: electric dipole or quadrupole? *X-Ray Spectrom.* **37**, 572-584 (2008).
11. Oishi, M. et al. Charge compensation mechanisms in $Li_{1.16}Ni_{0.15}Co_{0.19}Mn_{0.50}O_2$ positive electrode material for Li-ion batteries analyzed by a combination of hard and soft X-ray absorption near edge structure. *J. Power Sources* **222**, 45-51 (2013).
12. Qiao, R.M., Chuang, Y.D., Yan, S.S. & Yang, W.L. Soft X-Ray Irradiation Effects of Li2O2, Li_2CO_3 and Li_2O Revealed by Absorption Spectroscopy. *Plos One* **7** (2012).
13. Ruckman, M.W. et al. Interpreting the near edges of O_2 and O_2^- in alkali-metal superoxides. *Phys. Rev. Lett.* **67**, 2533-2536 (1991).
14. Hong, J. et al. Critical Role of Oxygen Evolved from Layered Li–Excess Metal Oxides in Lithium Rechargeable Batteries. *Chem. Mater.* **24**, 2692-2697 (2012).
15. Yabuuchi, N., Kubota, K., Aoki, Y. & Komaba, S. Understanding Particle-Size-Dependent Electrochemical Properties of Li_2MnO_3-Based Positive Electrode Materials for Rechargeable Lithium Batteries. *J. Phys. Chem. C* **120**, 875-885 (2016).
16. Lu, Y.C., Mansour, A.N., Yabuuchi, N. & Shao-Horn, Y. Probing the Origin of Enhanced Stability of "$AlPO_4$" Nanoparticle Coated LiCoO2 during Cycling to High Voltages: Combined XRD and XPS Studies. *Chem. Mater.* **21**, 4408-4424 (2009).
17. Yabuuchi, N., Tahara, Y., Komaba, S., Kitada, S. & Kajiya, Y. Synthesis and Electrochemical Properties of Li_4MoO_5-NiO Binary System as Positive Electrode Materials for Rechargeable Lithium Batteries. *Chem. Mater.* **28**, 416-419 (2016).
18. Sathiya, M. et al. Reversible anionic redox chemistry in high-capacity layered-oxide electrodes. *Nat. Mater.* **12**, 827-835 (2013).
19. Perdew, J.P., Burke, K. & Ernzerhof, M. Generalized gradient approximation made simple. *Phys. Rev. Lett.* **77**, 3865-3868 (1996).

20. Perdew, J.P. et al. Restoring the Density-Gradient Expansion for Exchange in Solids and Surfaces. *Phys. Rev. Lett.* **100**, 136406 (2008).
21. Blochl, P.E. PROJECTOR AUGMENTED-WAVE METHOD. *Phys. Rev. B* **50**, 17953-17979 (1994).
22. Hautier, G., Ong, S.P., Jain, A., Moore, C.J. & Ceder, G. Accuracy of density functional theory in predicting formation energies of ternary oxides from binary oxides and its implication on phase stability. *Phys. Rev. B* **85** (2012).
23. Shishido, T., Suzuki, H., Ukei, K., Hibiya, T. & Fukuda, T. Flux growth and crystal structure determination of trilithium niobate. *J. Alloys Compd.* **234**, 256-259 (1996).
24. Poon, P.W. & Carter, J.N. Genetic algorithm crossover operators for ordering applications. *Computers & Operations Research* **22**, 135-147 (1995).
25. Jay, E.E., Rushton, M.J.D., Chroneos, A., Grimes, R.W. & Kilner, J.A. Genetics of superionic conductivity in lithium lanthanum titanates. *PCCP* **17**, 178-183 (2015).
26. van de Walle, A. & Ceder, G. Automating first-principles phase diagram calculations. *J. Phase Equilib.* **23**, 348-359.
27. Walle, A.v.d. & Asta, M. Self-driven lattice-model Monte Carlo simulations of alloy thermodynamic properties and phase diagrams. *Modell. Simul. Mater. Sci. Eng.* **10**, 521 (2002).
28. van de Walle, A., Asta, M. & Ceder, G. The alloy theoretic automated toolkit: A user guide. *Calphad* **26**, 539-553 (2002).
29. van de Walle, A. Multicomponent multisublattice alloys, nonconfigurational entropy and other additions to the Alloy Theoretic Automated Toolkit. *Calphad* **33**, 266-278 (2009).
30. Hart, G.L.W. & Forcade, R.W. Algorithm for generating derivative structures. *Phys. Rev. B* **77**, 224115 (2008).
31. Zhou, F., Marianetti, C.A., Cococcioni, M., Morgan, D. & Ceder, G. Phase separation in Li_xFePO_4 induced by correlation effects. *Phys. Rev. B* **69** (2004).
32. Zhou, F., Cococcioni, M., Marianetti, C.A., Morgan, D. & Ceder, G. First-principles prediction of redox potentials in transition-metal compounds with LDA+U. *Phys. Rev. B* **70**, 235121 (2004).
33. Wang, L., Maxisch, T. & Ceder, G. Oxidation energies of transition metal oxides within the GGA+U framework. *Phys. Rev. B* **73**, 195107 (2006).
34. Jain, A. et al. Formation enthalpies by mixing GGA and GGA+U calculations. *Phys. Rev. B* **84**, 045115 (2011).
35. Miwa, K. & Fukumoto, A. First-principles study on $3d$ transition-metal dihydrides. *Phys. Rev. B* **65**, 155114 (2002).
36. Hoffmann, R. How Chemistry and Physics Meet in the Solid State. *Angew. Chem. Int. Ed.*

Engl. **26**, 846-878 (1987).

37. Dronskowski, R. & Bloechl, P.E. Crystal orbital Hamilton populations (COHP): energy-resolved visualization of chemical bonding in solids based on density-functional calculations. *J. Phys. Chem.* **97**, 8617-8624 (1993).
38. Deringer, V.L., Tchougréeff, A.L. & Dronskowski, R. Crystal Orbital Hamilton Population (COHP) Analysis As Projected from Plane-Wave Basis Sets. *J. Phys. Chem. A* **115**, 5461-5466 (2011).
39. Maintz, S., Deringer, V.L., Tchougréeff, A.L. & Dronskowski, R. Analytic projection from plane-wave and PAW wavefunctions and application to chemical-bonding analysis in solids. *J. Comput. Chem.* **34**, 2557-2567 (2013).
